# Effect of light on ascorbic acid biosynthesis and bioinformatics analysis of related genes in Chinese chives

**DOI:** 10.1371/journal.pone.0307527

**Published:** 2024-08-22

**Authors:** Yuxuan Qian, Jing Tong, Ning Liu, Baoju Wang, Yanhai Ji, Zhanhui Wu

**Affiliations:** 1 Beijing Vegetable Research Center, Beijing Academy of Agriculture and Forestry Science, Beijing, China; 2 Beijing University of Agriculture, Beijing, China; 3 Key Laboratory of Urban Agriculture (North) of Ministry of Agriculture and Rural Affairs, Beijing, China; ICAR-CPRI: Central Potato Research Institute, INDIA

## Abstract

Ascorbic acid (AsA) is an essential nutritional component and powerful antioxidant in vegetables, and in plants, AsA levels are regulated by light. AsA levels in the leaves of Chinese chive (*Allium tuberosum* Rottler ex Spr), a popular vegetable, are poorly understood. Thus, this study was performed to assess the influence of light on AsA biosynthesis in chive and select related genes (*AtuGGP1* and *AtuGME1*); in addition, bioinformatic analyses and gene expression level assays were performed. The biological information obtained for *AtuGGP1* and *AtuGME1* was analysed with several tools, including NCBI, DNAMAN, and MEGA11. After different light treatments were performed, the Chive AsA content and *AtuGGP1* and *AtuGME1* expression levels were determined. These results suggest that 1) compared with natural light, continuous darkness inhibited AsA synthesis in chives. 2) The amino acid sequences of *AtuGGP1* and *AtuGME1* are very similar to those of other plants. 3) The trends observed for the expression levels of *AtuGGP1* and *AtuGME1* were consistent with the AsA content observed in chives. Hence, we speculated that light controls AsA biosynthesis in chives by regulating *AtuGGP1* and *AtuGME1* expression. This study provided impactful and informative evidence regarding the functions of GGP and GME in chives.

## Introduction

Chinese chive (*Allium tuberosum* Rottler. ex Spr.), a perennial plant of the Allium genus, has been widely cultivated and naturalized worldwide [1]. Due to their high nutritional and medicinal properties, Chinese chives have become increasingly popular in food inventories as multifunctional vegetables; this popularity greatly reflects consumers’ awareness of the medicinal potential of chives to improve health and prevent chronic diseases [2–5].

Ascorbic acid (AsA), also known as vitamin C (VC), is a potent water-soluble antioxidant in humans and plays important roles in the synthesis of organic components within the extracellular matrix [6]. In plants, AsA acts as a major antioxidant and enzymatic cofactor that participates in several cellular and molecular processes [7, 8]. Several pathways for AsA biosynthesis have been identified, including the D-mannose/L-galactose pathway [9], D-galacturonate pathway [10], myoinositol pathway [11], and L-gulose pathway [12]. The L-galactose pathway has been identified as the primary pathway for AsA biosynthesis in many higher plants [13–17]. Gene expression levels in the AsA metabolic pathway are closely related to changes in AsA accumulation [18]. Several key genes involved in AsA accumulation have been identified in various plants. In this complex process, GDP-L-galactose phosphorylase (GGP) is a rate-limiting enzyme that plays a vital role in AsA synthesis and is a major determinant of AsA concentrations [19]. GGP is the first specific enzyme in the L-galactose pathway and catalyses the conversion of GDP-L-galactose into L-galactose-1-phosphate [20]. Additionally, GDP-mannose 3,5-epimerase (GME) synergistically controls AsA biosynthesis [21] and catalyses the epimerization of GDP-D-mannose, generating GDP-L-galactose in the L-galactose pathway and GDP-L-gulose in an alternative L-gulose pathway [22, 23]. The AsA content was increased by 1.5- to 2.5-fold by the overexpression of *GME* and *GGP* genes in the D-mannose/L-galactose pathway within Arabidopsis and rice leaves, which was achieved through the genetic manipulation of the AsA biosynthesis process [24, 25]. Previous studies revealed that the AsA content decreased significantly in *GME*-silenced tomatoes [26]. Among the *GGP* deletion mutants, the AsA content in tomato fruit was significantly reduced [27].

Light is essential for the activation of AsA biosynthesis [28, 29], and research has shown that light induces many genes in the AsA synthesis pathway (such as *GMP*, *GME*, *GGP*, *GPP*, *GalDH*, and *GLDH*) that contain optical response elements on their promoters [30]. Under dark conditions, the transcription levels of *GGP* and *GME* were decreased, and the AsA content in Arabidopsis leaves decreased by 91%; however, after prolonged illumination, the AsA content was 1.71 times greater than that in the control [28]. Shading leads to a decrease in the expression level of most genes that synthesize AsA, of which *GME* and *GGP* are the most reduced [31]. Compared with the conventional optical period (12 h/12 h), continuous light (24 h/0 h) significantly increased the content of AsA in lettuce leaves [32]. Continuous light also gradually upregulated the expression of *GME* and *GGP* genes [28].

Here, we show that plant *GGP* and *GME* expression levels are largely influenced by light, which thus affects AsA biosynthesis. However, studies on how light affects AsA synthesis and *GGP* and *GME* expression in chives are scarce. Therefore, this study was performed to determine the physicochemical properties of GGP and GME in chive by bioinformatic analysis, as well as the influence of light on the AsA content and *GGP* and *GME* expression levels in chives, to provide more impactful and informative information on the functions of GGP and GME in chive.

## Materials and methods

### Plant materials

Seeds of the *A*. *tuberosum* cultivar ‘791’ were germinated on substrates (peat: vermiculite = 3: 1) in a balcony greenhouse located in the Beijing Vegetable Research Center, Beijing Academy of Agriculture and Forestry Sciences (latitude: 39°56′18′′N; longitude: 116°17′03′′E). After the first crop harvest, the chives were treated as follows: 1) the plants were grown under natural light (L) or continuous darkness (D). Fresh leaves of plants were collected after 10 days, 20 days, 30 days and 40 days under treatments (samples were used in three replicates). 2) The plants were grown under the following photoperiods: 0 d (40 d continuous dark), 10 d (30 d continuous dark + 10 d natural light), 20 d (20 d continuous dark + 20 d natural light), 30 d (10 d continuous dark + 30 d natural light), and 40 d (40 d natural light), and fresh leaves were collected (samples were used in three replicates). The plants in each treatment group were irrigated with nutrient solution every 3 days. The composition was as follows: Ca(NO_3_)_2_ 1.0 mM, KNO_3_ 4.0 mM, NH_4_NO_3_ 2.0 mM, KH_2_PO_4_ 2.0 mM, (NH_4_)_2_SO_4_ 1.0 mM, MgSO_4_·7H_2_O 1.0 mM [33]. All the collected samples were immediately frozen in liquid nitrogen and stored at -80°C before use.

### Total RNA isolation and first-strand cDNA synthesis

Total RNA from the chive leaves was extracted using a Vazyme FastPure Universal Plant Total RNA Isolation Kit (Nanjing Novizan Biotechnology Co., Ltd.) in accordance with the manufacturer’s instructions. Total RNA was dissolved in 100 μL of RNase-free water, and the concentrations were measured with a NanoDrop 2000 spectrophotometer (Thermo Fisher Scientific, Waltham, MA, USA). cDNA was synthesized using the TRAN EasyScript One-Step gDNA Removal and cDNA Synthesis SuperMix Kit (Beijing All-style Gold Biotechnology Co., Ltd.). Briefly, total RNA was mixed with dNTPs in a total volume of 20 μL and incubated for 5 min at 65°C, 30 min at 42°C, or 5 s at 85°C in accordance with the manufacturer’s instructions. First-strand cDNA was stored at -20°C before use.

### Cloning the full-length cDNA of *AtuGGP1* and *AtuGME1*

The transcriptome sequencing results revealed one homologous *GGP* gene sequence and one homologous *GME* gene sequence, which contained complete open reading frames (ORFs). We named them *AtuGGP1* (387 bp) and *AtuGME1* (1140 bp). The specific primers *AtuGGP1*-FP/*AtuGGP1*-RP and *AtuGME1*-FP/*AtuGME1*-RP were designed for these two genes (Table 1) using SnapGene 7.0.2.0 to amplify the cDNA sequences of *AtuGGP1* and *AtuGME1*. First-strand cDNA was used as a template to amplify the cDNA of *AtuGGP1* and *AtuGME1* with 2×A8 PCR Mix (Aidlab Biotechnologies Co., Ltd., Beijing, China) according to the manufacturer’s instructions. The PCR products were purified, cloned and inserted into the *pEASY*^®^-Blunt E2 vector (TransGen Biotech, Beijing, China), and sequenced by Shanghai Sangon Biotech Co., Ltd. (Shanghai, China).

**Table 1:** Primer sequences for *AtuGGP1* and *AtuGME1*.

Primers	Sequence (5’–3’)
*AtuGGP1*-FP	ATGATAAGAACTAAAGTCGGGGAC
*AtuGGP1*-RP	TCACTGGAGAACAATACAACCTTC
*AtuGME1*-FP	ATGGGTGATAACTCGGACAAAAC
*AtuGME1*-RP	TTAGAGCCCTTCATTTCCATCAG

### Bioinformatic analysis

The protein physicochemical properties were analysed by ProtPatham, and the secondary structures were predicted by NovoPro. Both ProtPatham and NovoPro were used to analyse the protein hydrophilicity. The Molecular Bioinformatics Center was used to predict protein subcellular localization. The protein phosphorylation sites were predicted using NetPhos 3.1. Finally, amino acid homologous sequence alignment and phylogenetic tree construction were completed using NCBI BLAST, DNAMAN and MEGA11 (Table 2).

**Table 2:** Bioinformatics analysis software and website.

Software or website	Function
ProtParam, ProtScale (https://www.ExPASy.org/)	Protein physicochemical property and hydrophilicity analysis
NovoPro Net (https://novopro.cn/tools/)	Secondary structure prediction, hydrophilicity analysis
Molecular Bioinformatics Center (http://cello.life.nctu.edu.tw/)	Subcellular localization prediction
NetPhos 3.1 (https://services.healthtech.dtu.dk/services/NetPhos-3.1/)	Protein phosphorylation site prediction
NCBI, BLAST (https://blast.ncbi.nlm.nih.gov/)	Comparing amino acid homologous sequence; Derderive the amino acid sequence
DNAMAN	Amino acid multiple sequence alignment
MEGA11	Building phylogenetic evolutionary trees

### Analyses of *AtuGGP1* and *AtuGME1* expression by real-time quantitative PCR

Real-time quantitative PCR cDNA amplification was carried out on a Bio-Rad CFX Opus 96 real-time PCR system (CFX Opus 96, USA). SYBR Green Mix (2×) (Toyobo (Shanghai) Biotech Co., Ltd.) was used in all reactions according to the protocol described by the manufacturer. Gene-specific primers were designed using Primer Premier 5.0 software (Table 3). The volume of each reaction was 20 μL (10.0 μL of SYBR Green Mix (2×), 0.5 μL of forwards and reverse primer each, 2.0 μL of cDNA, and 7.0 μL of RNase-free ddH_2_O). *AtuSKP1*/*DN253_c0_g1* was selected as the housekeeping gene, and the relative expression levels were calculated using the 2^-△△Ct^ method. Three biological replicates were performed.

**Table 3:** Primer sequences and reaction program for qRT‒PCR analysis.

Primer name	Primer sequence (5’-3’)	Reaction program
*AtuSKP1*-F	GGGATGCCGATTTTGTTAAA	95°C (3 min); 95°C (10 s); 56°C (10 s); 72°C (30 s); followed by 44 cycles; 95°C (10 s)
*AtuSKP1*-R	TTTCCGGATCTCTTCTGGTG	
*AtuGGP1*-FQ	TTGGGAGATAAGCGGTCA	
*AtuGGP1*-RQ	ATGCCTGTAGCCTCGAAA	
*AtuGME1*-FQ	GTCGCAACTCTGACAACAC	
*AtuGME1*-RQ	TAAATAGACAAATCCACCCCT	

### Extraction and determination of AsA

Ascorbic acid oxidase (AAO) catalyses the oxidation of AsA to produce dehydroascorbic acid (DHA) [34]. Thus, the AsA content can be calculated by measuring the percentage of AAO that oxidizes AsA [35]. Briefly, plant leaves (0.1 g) were mixed with trichloroacetic acid (TCA) solution (1 mL) for homogenization and centrifuged at 8000 × g for 20 min. Afterwards, the supernatants were mixed with AAO. Then, the absorbances were measured after 10 s and 130 s by a spectrophotometer at a wavelength of 265 nm, and three biological and technical replicates were conducted.

### Data analysis

The significance of the differences in the data were assessed by PASW Statistics 18 by making multiple comparisons, and the charts were drawn with Excel 2016 software.

## Results

### Full-length cDNA cloning

**Fig 1:**
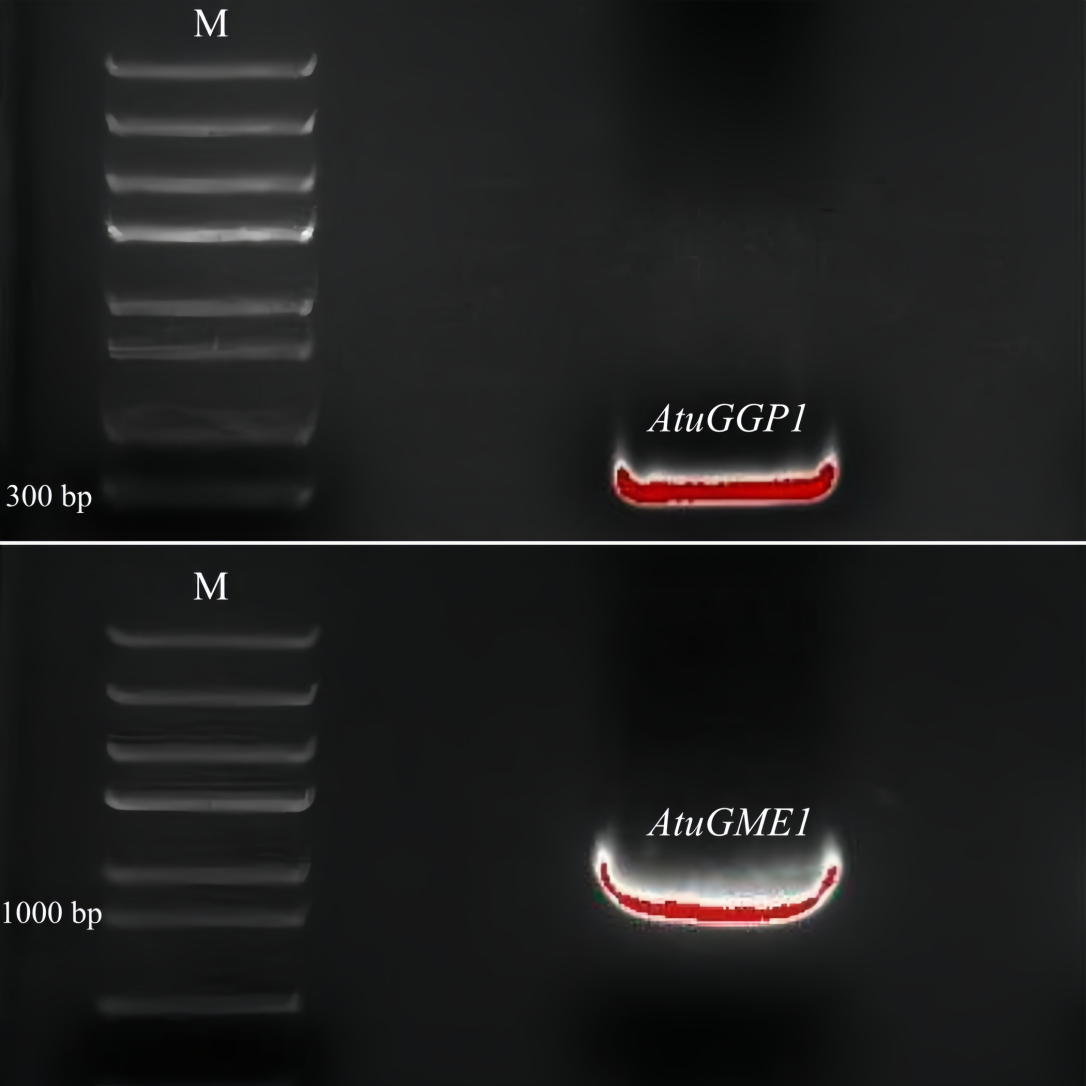


**Fig 2:**
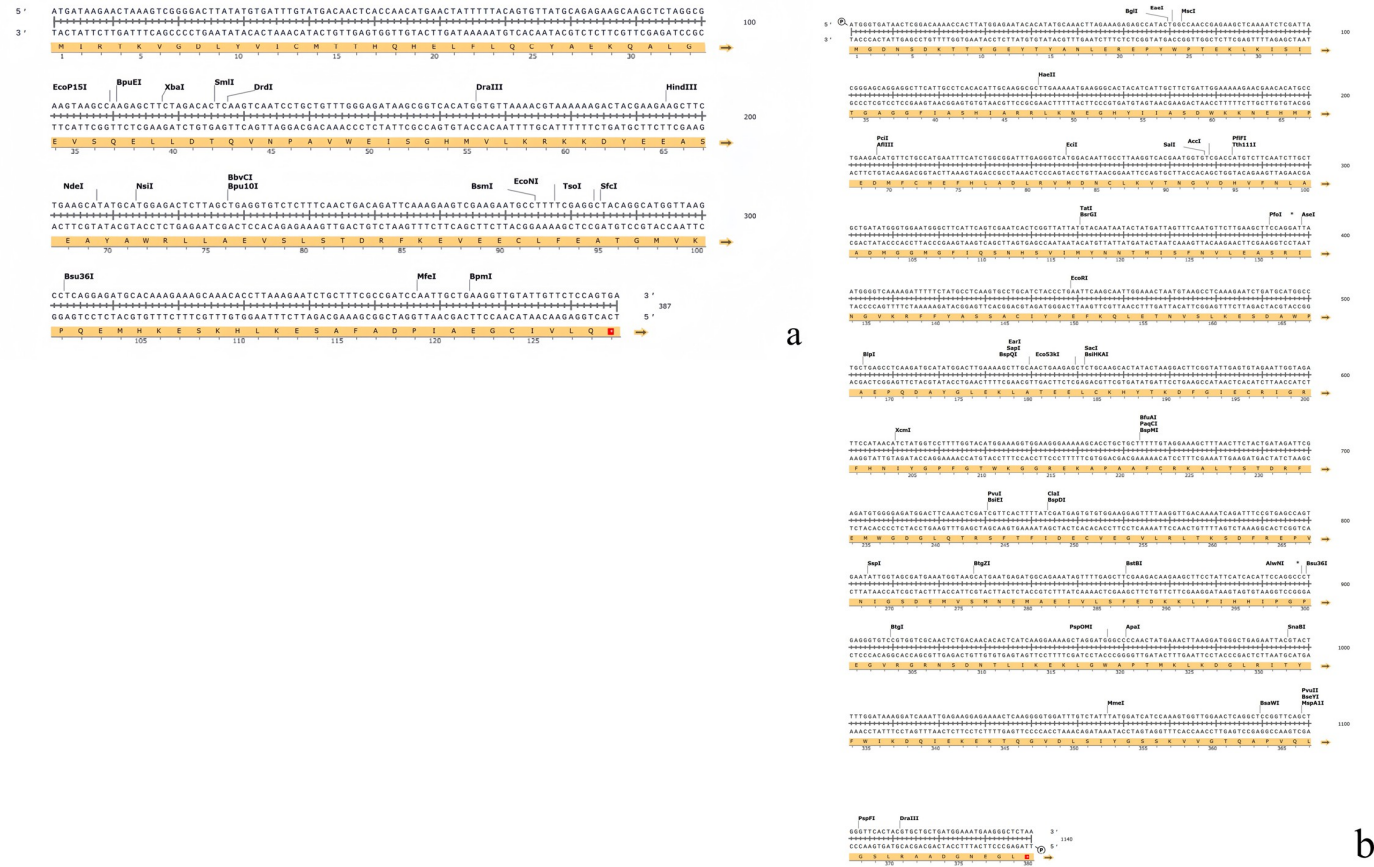


With the cDNA of the leaf as the template, the ORFs of *AtuGGP1* and *AtuGME1* were amplified via PCR to obtain two specific fragments. Agarose electrophoresis of the PCR products revealed that *AtuGGP1* had a single band at approximately 300 bp, *AtuGME1* had a single band at approximately 1000 bp, and there were no nonspecific amplification bands (Fig 1). These fragments were linked with the *pEASY*^®^-Blunt E2 vectors, and the positive clones were selected. After sequencing and comparison were performed, the sequences of *AtuGGP1* and *AtuGME1* were confirmed. According to the amino acid sequence inferred from SnapGene 7.0.2.0, the ORF of *AtuGGP1* is 387 bp, encoding a total of 128 amino acids, and that of *AtuGME1* is 1140 bp, encoding a total of 379 amino acids (Fig 2).(Data shown in S1 File)

### Bioinformatic analysis

The protein physicochemical properties of *AtuGGP1* and *AtuGME1* were analysed using ProtPatham, and the results showed that the relative molecular weight of the *AtuGGP1* protein was 14676.84 D, the molecular formula was C_650_H_1024_N_170_O_198_S_9_, the aliphatic index was 86.09, the theoretical isoelectric point was 5.09 (<7), and the instability coefficient was 43.77 (>40), indicating that *AtuGGP1* is an unstable acidic protein. The relative molecular weight of the *AtuGME1* protein was 42804.56 D, the molecular formula was C_1903_H_2936_N_516_O_570_S_20_, the aliphatic index was 72.59, the theoretical isoelectric point was 5.83 (<7), and the instability coefficient was 39.00 (<40), indicating that *AtuGME1* is a stable acidic protein (Table 4).

**Table 4:** Physical and chemical properties of *AtuGGP1* and *AtuGME1*.

Genes	Molecular Weight/D	pI	Instability Index	Grand Average of Hydropathicity	Aliphatic Index	The High Content of Amino Acid
*AtuGGP1*	14676.84	5.09	43.77	-0.291	86.09	Glu 13.3% Leu 10.2% Ala 8.6%
*AtuGME1*	42804.56	5.83	39.00	-0.445	72.59	Gly 8.7% Glu 8.2% Leu 7.1% Lys 7.1%

**Fig 3:**
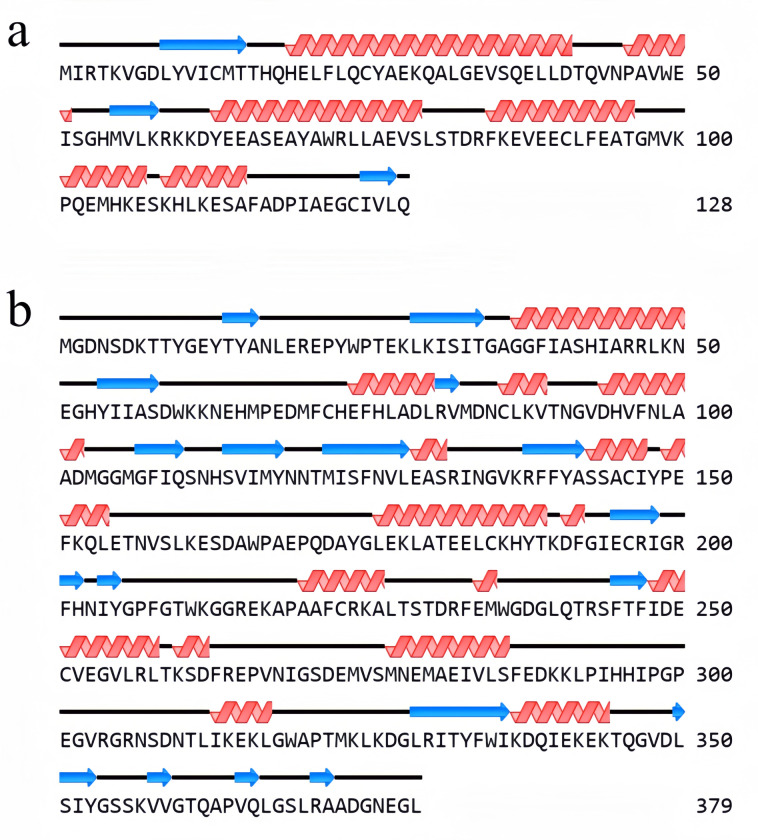


**Fig 4:**
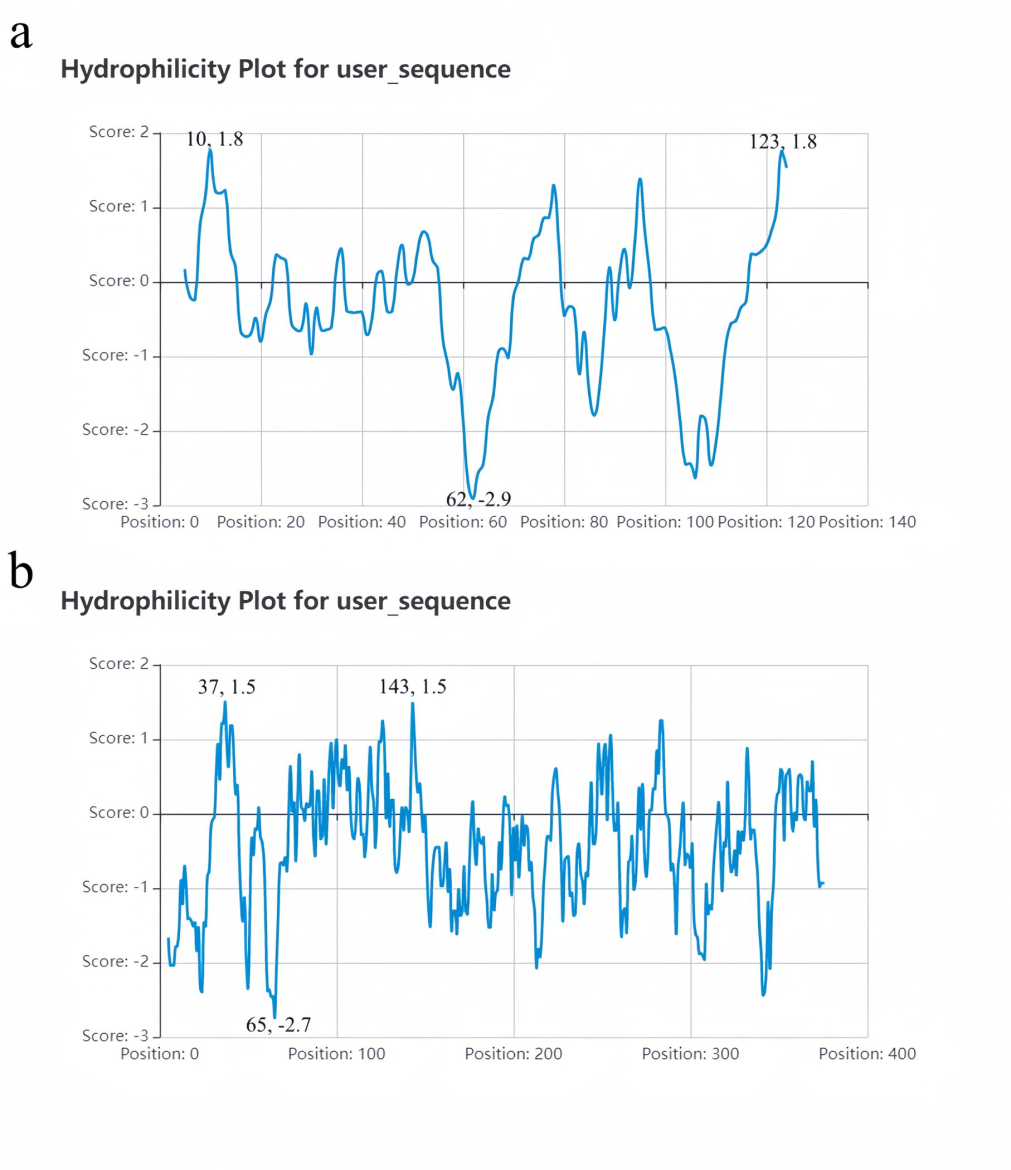


The protein secondary structures of *AtuGGP1* and *AtuGME1* were predicted by NovoPro. The results showed that the α-helix, extended chain, and irregular curl of the *AtuGGP1* protein accounted for 56.25%, 10.94% and 32.81% of the structure, respectively. The α-helix, extended chain, and irregular curl of the *AtuGME1* protein accounted for 31.66%, 17.41% and 50.92% of the structure, respectively (Fig 3). The protein hydrophilicities of *AtuGGP1* and *AtuGME1* were analysed via ProtPatham and NovoPro, and the results indicated that the maximum hydrophobicity of *AtuGGP1* was 1.8 (at positions 10 and 123), the minimum hydrophobicity was -2.9 (at position 62), and the average hydrophilicity was -0.291, suggesting that *AtuGGP1* is a hydrophilic protein. The maximum hydrophobicity of *AtuGME1* is 1.5 (at positions 37 and 143), the minimum hydrophobicity is -2.7 (at position 65), and the average hydrophilicity is -0.445, suggesting that *AtuGME1* is a hydrophilic protein (Table 4; Fig 4). Molecular Bioinformatics Center online software predicted that the *AtuGGP1* and *AtuGME1* proteins are most likely located in the cytoplasm.

**Fig 5:**
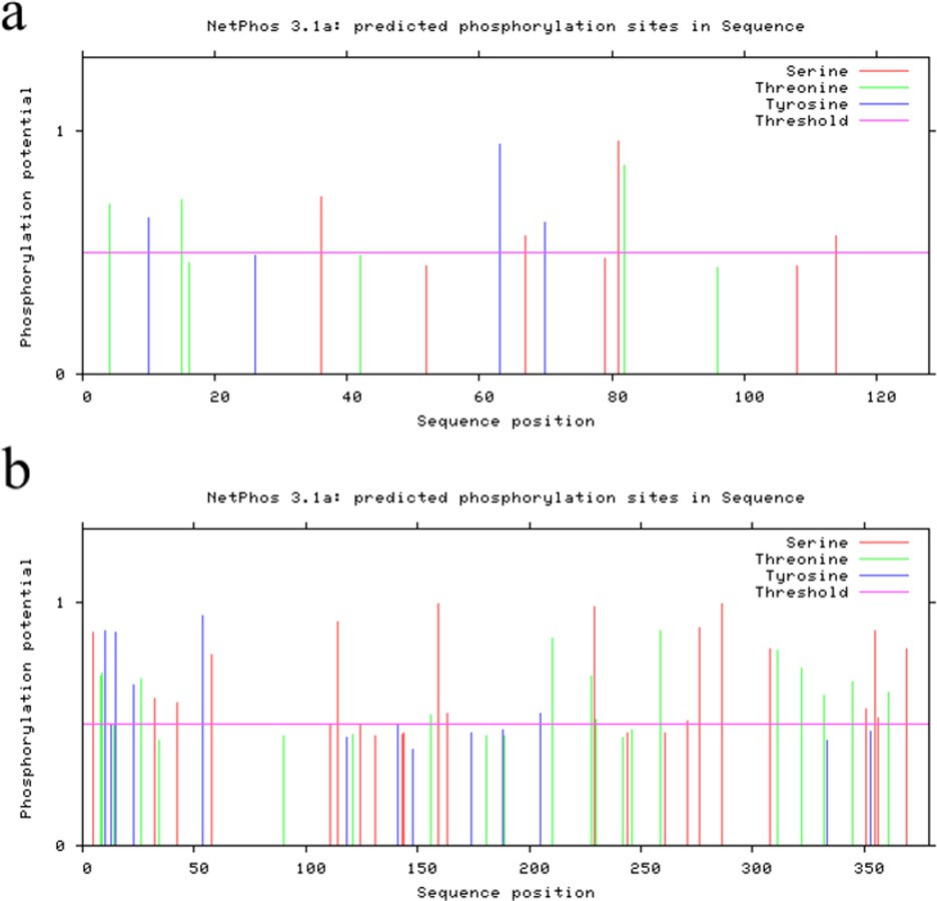


Phosphorylation affects protein activity and is closely related to signal transmission. NetPhos 3.1 predicted that the *AtuGGP1* protein contains 10 highly reliable phosphorylation sites, including 4 serine, 3 threonine, and 3 tyrosine residues. The *AtuGME1* protein contains 35 highly reliable phosphorylation sites, including 16 serine, 13 threonine, and 6 tyrosine residues (Fig 5). It is speculated that *AtuGGP1* and *AtuGME1* are mainly composed of serine and other amino acids that act as auxiliaries, completing phosphorylation and regulating biological functions.

**Fig 6:**
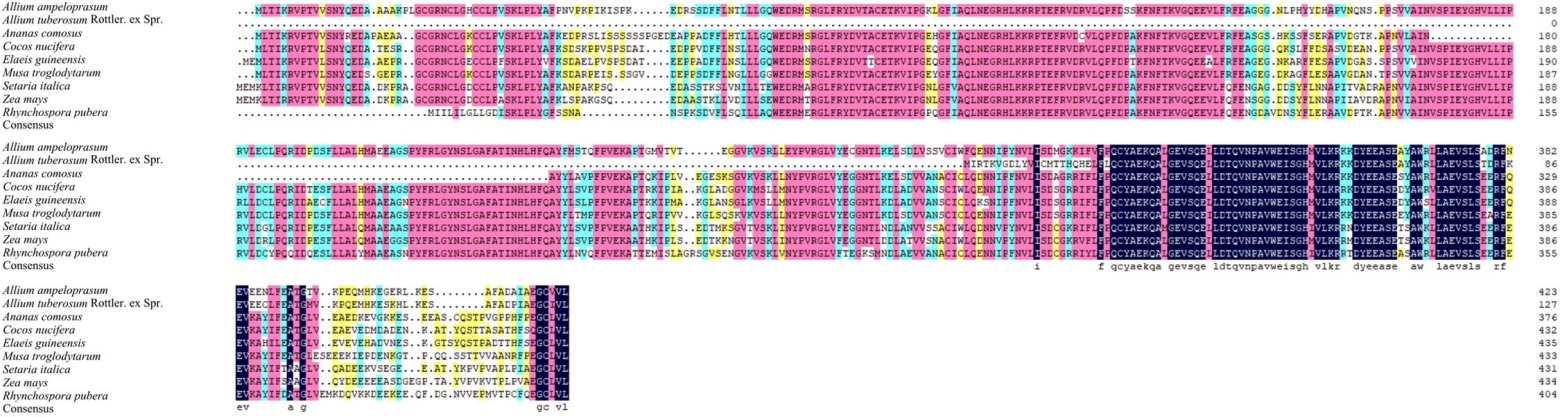


**Fig 7:**
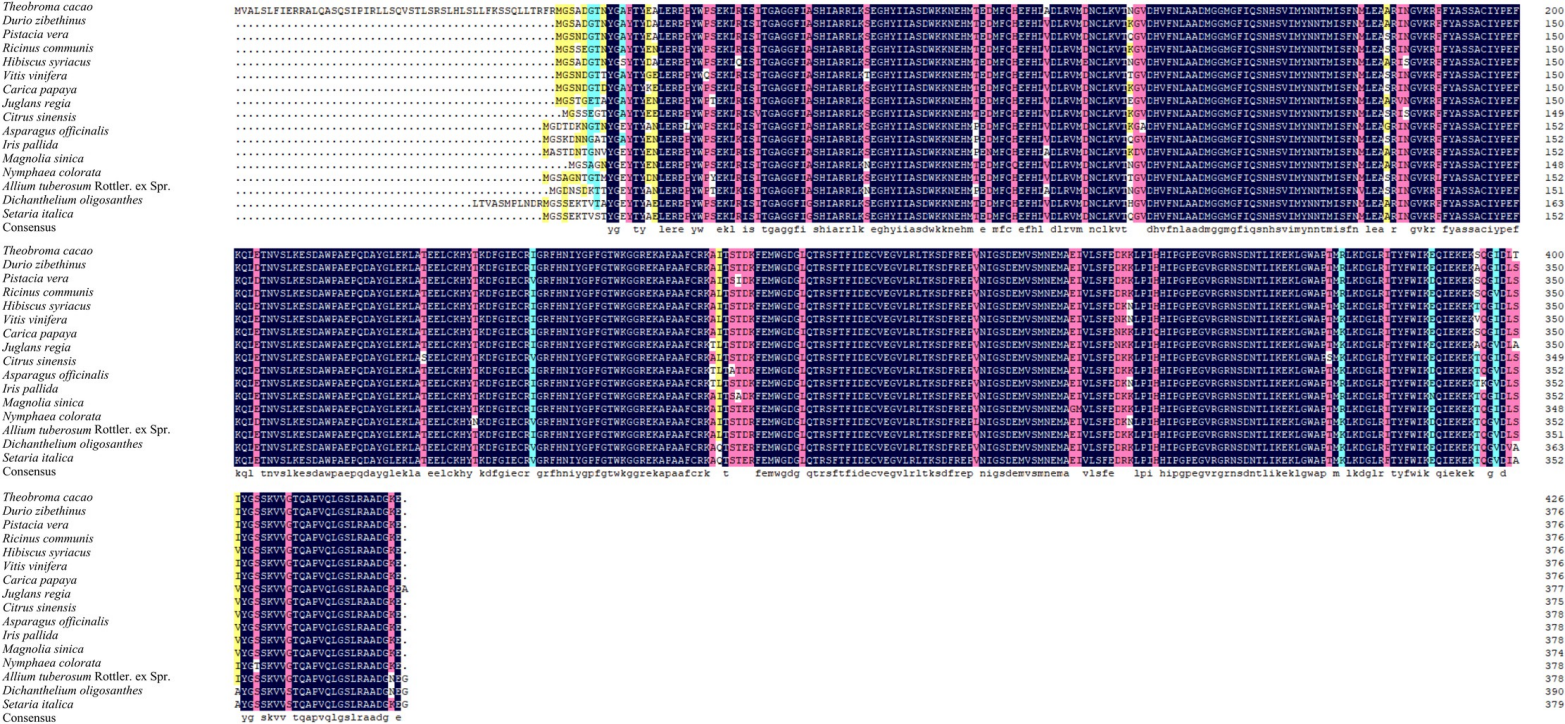


**Fig 8:**
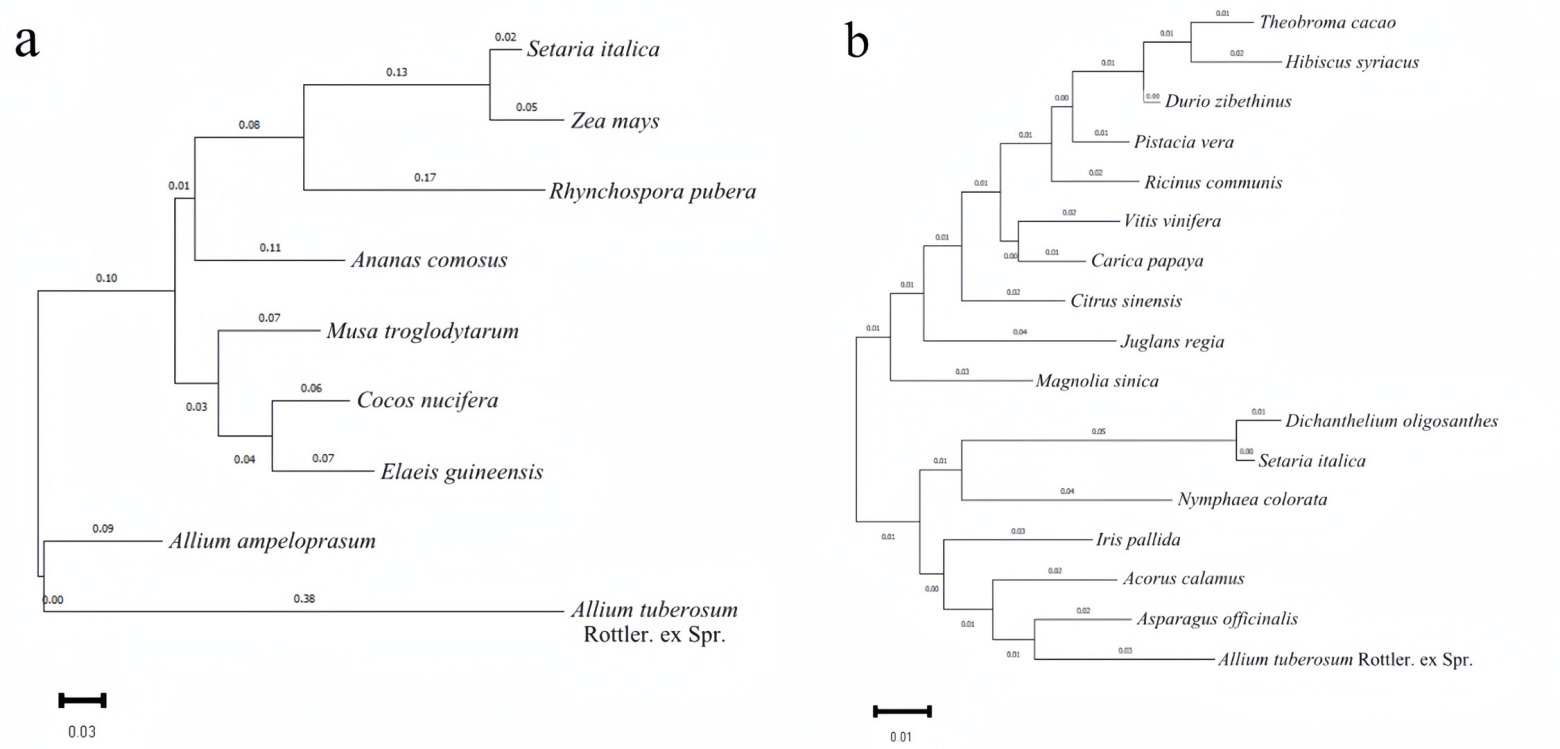


The amino acid sequences encoded by *AtuGGP1* and *AtuGME1* were used to select homologous sequences from other plants. These results suggest that the amino acid sequences of *AtuGGP1* and *AtuGME1* are highly similar to those of other plants (Figs 6 and 7). The amino acid sequence of *AtuGGP1* is highly homologous to that of 8 other plant species, including blue leek, pineapple, coconut, plantain, and oil palm, at over 50%. The amino acid sequence of *AtuGME1* is highly homologous to that of 16 plant species, including asparagus, Calamus, Iris pallida, and millet, which are more than 90% homologous. Based on the *AtuGGP1* and *AtuGME1* protein sequences, phylogenetic trees were constructed using MEGA11 software. Based on the phylogenetic tree analyses, *AtuGGP1* was not closely related to plants such as oil palm, coconut, and plantain, and blue leek contained the most closely related gene to *AtuGGP1*. The plant with the most closely related gene to *AtuGME1* was asparagus, followed by calamus and Iris pallida; in contrast, plants such as Magnolia sinica, papaya and pistachio had relatively distant relationships with *AtuGME1* (Fig 8).

### AsA content and gene expression level

**Fig 9:**
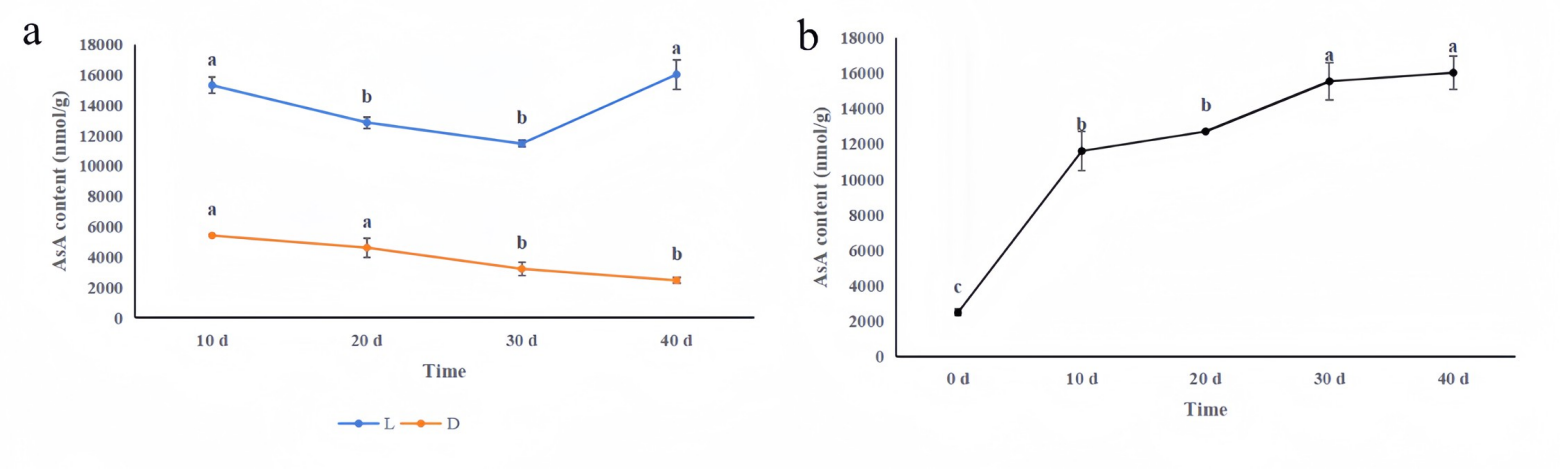


As shown in Fig 9A, the AsA content gradually decreased as the chives grew in natural light but gradually increased after 30 d. When the chives grew in the dark, the AsA content gradually decreased. On the 30th and 40th days, the chive AsA content was significantly lower than that on the 10th and 20th days. During the 40-day growth period, the AsA content was greater in the chives that grew in natural light that in the chives that grew in continuous darkness.

The results in Fig 9B show that different proportions of lighting time influenced the chive AsA content during the 40-day growth period. The content of AsA gradually increased with increasing natural lighting time. After 10 d, 20 d, 30 d and 40 d of natural lighting, the chive AsA content significantly increased to 370.6%, 415.4%, 530.4% and 550.2% greater than that after 0 d of natural lighting, respectively (Data shown in S1 Table).

**Fig 10:**
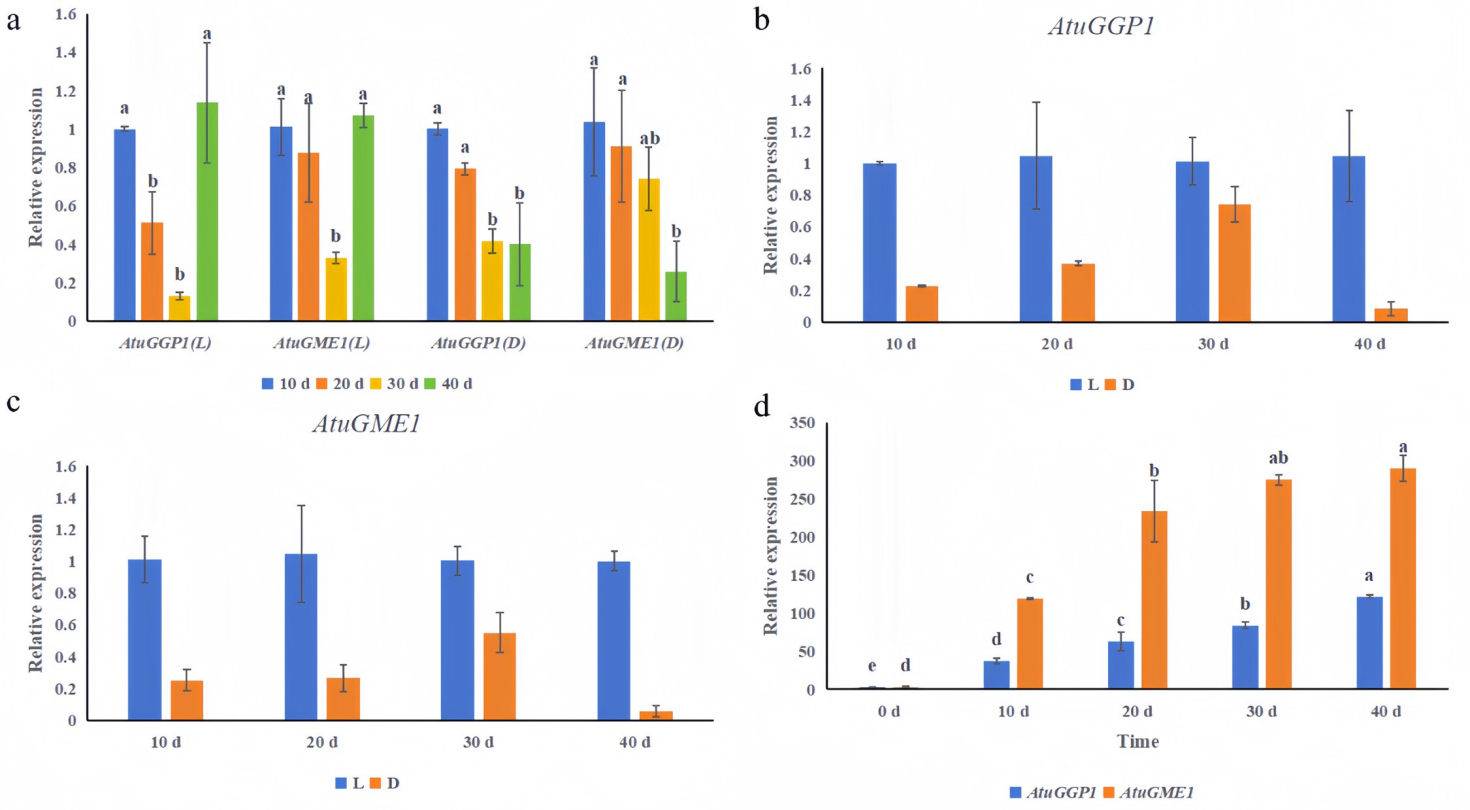


These results indicate that light affects *AtuGGP1* and *AtuGME1* expression levels. When chives were grown in natural light, *AtuGGP1* and *AtuGME1* expression levels gradually decreased, but after 30 d, the levels gradually increased (Fig 10A). As the chives grew in the dark, *AtuGGP1* and *AtuGME1* expression levels gradually decreased (Fig 10A). Thus, the change in AsA content in chives was consistent with the changes in *AtuGGP1* and *AtuGME1* expression levels. Additionally, compared with those in chives growing in natural light, *AtuGGP1* and *AtuGME1* expression levels in chives growing in continuous darkness were lower during the 40-day growth period (Fig 10B and 10C). Moreover, different proportions of lighting time influenced *AtuGGP1* and *AtuGME1* expression levels, which gradually increased with increasing lighting time; this result showed that the tendency of the AsA content in chives with different durations of lighting was consistent with that of the *AtuGGP1* and *AtuGME1* expression levels (Fig 10D). Thus, we can infer that *AtuGGP1* and *AtuGME1* affect AsA biosynthesis in chives (Data shown in S2 Table).

## Discussion

AsA is among the essential components of fruit and vegetable nutritional quality [30]. Many horticultural plants contain high levels of AsA; for example, the AsA content in mature fruits of prickly pear, hawthorn, rosehip, and cherokee rose-hip can reach 800 mg per 100 g. The AsA content is usually higher in wild fruits than in cultivated fruits [36]. AsA is a powerful antioxidant that may decrease the incidence of several illnesses, such as cancer and cardiovascular diseases [37]; however, the human body cannot synthesize AsA because of a lack of the L-guluronic acid-1,4-lactone oxidase enzyme [38]. Thus, plant-derived foods are the main source of AsA for humans. Furthermore, AsA is an essential antioxidant in plants that scavenges reactive oxygen species (ROS) generated during exposure to biotic or abiotic stresses and those produced during normal growth and development [7, 39, 40].

Chinese chives are cultivated in China and other Asian countries as well as many European countries because this popular vegetable is rich in vitamins, fibre, and sulfur compounds and exhibits antibiotic properties [41, 42]. Although these nutritional qualities are well known, knowledge regarding AsA compounds in the leaves of Chinese chives is lacking. Light is an important signal for the activation of plant AsA biosynthesis, and researching the effects of light on AsA biosynthesis is convenient and inexpensive. Hence, we assessed the influence of light on AsA biosynthesis and selected related genes in chives; performed bioinformatic analyses; and performed assays to investigate the levels of gene expression in chive.

There are four possible AsA biosynthetic pathways in plants: the L-galactose, L-gulose, myo-inositol, and D-galacturonic acid pathways. The L-galactose pathway seems to prevail in many species [43]. First, 6-phosphate-D-glucose undergoes a series of isomerization and translocation reactions to produce 1-phosphate-D-mannose, after which GDP-D-mannose pyrophosphatase (GMP/VTC1) catalyses the transformation of 1-phosphate-D-mannose to GDP-D-mannose [44]. Second, GDP-D-mannose is catalysed by GME to produce GDP-L-galactose or GDP-L-gulose, and then the GGP catalyses GDP-L-galactose to form 1-phosphate-L-galactose [45]. Third, L-galactose-1-phosphatase (GPP) and L-galactose dehydrogenase (GalDH) catalyse the formation of L-galactose from 1-phosphate-L-galactose and L-galactono-1,4-lactone, respectively [46].

L-galactono-1,4-lactone dehydrogenase (GalLDH) is involved in the final step of the L-galactose pathway and catalyses the formation of AsA from L-galactono-1,4-lactone. Previous studies revealed that the overexpression of *GME* in tomato plants increased the AsA content and plant tolerance to salt, drought, and low temperatures [47, 48]. In contrast, after two *GME* genes in tomatoes were deleted, the AsA content decreased, the stems became brittle, and the fruit hardness decreased [26]. Tomato *GGP1* is a homologous gene of Arabidopsis *VTC2* and *VTC5*, and inhibiting *GGP1* expression can reduce fruit cold resistance and yield [27]. The simultaneous overexpression of *GME* and *GGP* significantly increased the AsA content in cherry fruits [49]. There was a significant correlation between the expression level of the *GGP* gene and the AsA content in kiwifruit leaves and fruits during the growth period [14]. *GGP* and *GME* play vital roles in plant AsA synthesis and significantly influence AsA content. Our results showed that the change in AsA content in chives was consistent with the changes in the expression levels of *AtuGGP1* and *AtuGME1* (Figs 9 and 10), which corresponds with previous studies on tomato [47] and kiwifruit [14]. *AtuGGP1* and *AtuGME1* might strongly influence AsA biosynthesis in chives.

Light exerts a regulatory effect on AsA levels in plants by stimulating the L-galactose pathway, inducing gene expression, and increasing plant AsA content via a series of important light signal transcription factors and proteins [50]. *GGP* and *GME* contain many optical response elements in their promoters [30]. For example, the Rosa roxburghii *GGP* promoter contains photoinduction elements such as ACE, AE-box, Box4, CAG-motif, G-Box, GA-motify, GAG-motif, GTI-motif and GATA-motif. Under continuous light treatment, most rosa roxburghii *GGP* promoter mutants showed a significant increase in activity, but their activity decreased to varying degrees under continuous dark treatment [51].

Early studies revealed that the AsA content in Arabidopsis leaves decreased by 40% after 24 h of dark treatment [52], while under high light conditions, the AsA content in kiwifruit fruits [53], tomato leaves and fruits [54], apple fruits [55], and cowpea seedlings [56] significantly increased. Under shading, the AsA content of tomato fruits decreased by 30%, and the *GME* and *GGP* expression levels decreased concurrently [31]. Our research revealed that the AsA content of chives grown in natural light was greater than that of chives grown in continuous darkness during the 40-day growth period (Fig 9A). In addition, as the lighting duration increased, the chive AsA content gradually improved (Fig 9B). The trends in the expression levels of *AtuGGP1* and *AtuGME1* were consistent with the abovementioned trends in AsA content (Figs 9 and 10). Therefore, we speculate that *AtuGGP1* and *AtuGME1* control chive AsA biosynthesis via optical response elements in their promoters.

The GGP protein plays a role in AsA synthesis and may function as a growth regulation factor. Research on Arabidopsis *vtc2* mutants revealed that the VTC2:GUS fusion protein is located simultaneously in the cytoplasm and nucleus; these results suggest that *GGP* plays an enzymatic role in the cytoplasm and performs specific functions to regulate gene expression in the nucleus [57]. Similarly, the tomato SlGGP-GFP fusion protein is localized in both the cytoplasm and nucleus of onion cells [58]. Additionally, previous research has shown that plants might experience slower cell division and growth due to the inactivation of GME [59], suggesting that *GME* controls plant growth. In addition to AsA synthesis, the functions of *GGP* and *GME* should be further studied.

In conclusion, light exerts a regulatory effect on AsA biosynthesis in chives. GGP and GME play vital roles in AsA synthesis, as they are light-induced and control plant AsA synthesis. The amino acid sequences of *AtuGGP1* and *AtuGME1* are very similar to those of other plants. AsA synthesis was inhibited in chives grown in continuous darkness compared to chives grown in natural light. Moreover, the trends in *AtuGGP1* and *AtuGME1* expression were generally consistent with the changes in AsA content in chives. These results suggest that light controls AsA biosynthesis in chives by regulating *AtuGGP1* and *AtuGME1* expression.

## Supporting information

S1 File AtuGGP1 and AtuGME1 CDS and amino acid sequences. (DOCX)

S1 Table AsA content of Chinese chive. There are three biological replicates.
(XLSX)

S2 Table AtuGGP1 and AtuGME1 expression levels. There are three biological replicates.
(XLSX)

S1 Fig Raw images of this article. (PDF)
