# Supplementary material for: Effect of light on ascorbic acid biosynthesis and bioinformatics analysis of related genes in Chinese chives
Source: PLoS One. 2024 Aug 22;19(8):e0307527. doi: 10.1371/journal.pone.0307527 (PMC11340962; doi:10.1371/journal.pone.0307527)
Supplement: S1 File — (DOCX) [file pone.0307527.s001.docx]

**GDP-L-galactose phosphorylase 1 (GGP1)**

ATGATAAGAACTAAAGTCGGGGACTTATATGTGATTTGTATGACAACTCACCAACATGAACTATTTTTACAGTGTTATGCAGAGAAGCAAGCTCTAGGCGAAGTAAGCCAAGAGCTTCTAGACACTCAAGTCAATCCTGCTGTTTGGGAGATAAGCGGTCACATGGTGTTAAAACGTAAAAAAGACTACGAAGAAGCTTCTGAAGCATATGCATGGAGACTCTTAGCTGAGGTGTCTCTTTCAACTGACAGATTCAAAGAAGTCGAAGAATGCCTTTTCGAGGCTACAGGCATGGTTAAGCCTCAGGAGATGCACAAAGAAAGCAAACACCTTAAAGAATCTGCTTTCGCCGATCCAATTGCTGAAGGTTGTATTGTTCTCCAGTGA

MIRTKVGDLYVICMTTHQHELFLQCYAEKQALGEVSQELLDTQVNPAVWEISGHMVLKRKKDYEEASEAYAWRLLAEVSLSTDRFKEVEECLFEATGMVKPQEMHKESKHLKESAFADPIAEGCIVLQ

**GDP-mannose 3,5-epimerase 1 (GME1)**

ATGGGTGATAACTCGGACAAAACCACTTATGGAGAATACACATATGCAAACTTAGAAAGAGAGCCATACTGGCCAACCGAGAAGCTCAAAATCTCGATTACGGGAGCAGGAGGCTTCATTGCCTCACACATTGCAAGGCGCTTGAAAAATGAAGGGCACTACATCATTGCTTCTGATTGGAAAAAGAACGAACACATGCCTGAAGACATGTTCTGCCATGAATTTCATCTGGCGGATTTGAGGGTCATGGACAATTGCCTTAAGGTCACGAATGGTGTCGACCATGTCTTCAATCTTGCTGCTGATATGGGTGGAATGGGCTTCATTCAGTCGAATCACTCGGTTATTATGTACAATAATACTATGATTAGTTTCAATGTTCTTGAAGCTTCCAGGATTAATGGGGTCAAAAGATTTTTCTATGCCTCAAGTGCCTGCATCTACCCTGAATTCAAGCAATTGGAAACTAATGTAAGCCTCAAAGAATCTGATGCATGGCCTGCTGAGCCTCAAGATGCATATGGACTTGAAAAGCTTGCAACTGAAGAGCTCTGCAAGCACTATACTAAGGACTTCGGTATTGAGTGTAGAATTGGTAGATTCCATAACATCTATGGTCCTTTTGGTACATGGAAAGGTGGAAGGGAAAAAGCACCTGCTGCTTTTTGTAGGAAAGCTTTAACTTCTACTGATAGATTCGAGATGTGGGGAGATGGACTTCAAACTCGATCGTTCACTTTTATCGATGAGTGTGTGGAAGGAGTTTTAAGGTTGACAAAATCAGATTTCCGTGAGCCAGTGAATATTGGTAGCGATGAAATGGTAAGCATGAATGAGATGGCAGAAATAGTTTTGAGCTTCGAAGACAAGAAGCTTCCTATTCATCACATTCCAGGCCCTGAGGGTGTCCGTGGTCGCAACTCTGACAACACACTCATCAAGGAAAAGCTAGGATGGGCCCCAACTATGAAACTTAAGGATGGGCTGAGAATTACGTACTTTTGGATAAAGGATCAAATTGAGAAGGAGAAAACTCAAGGGGTGGATTTGTCTATTTATGGATCATCCAAAGTGGTTGGAACTCAGGCTCCGGTTCAGCTGGGTTCACTACGTGCTGCTGATGGAAATGAAGGGCTCTAA

MGDNSDKTTYGEYTYANLEREPYWPTEKLKISITGAGGFIASHIARRLKNEGHYIIASDWKKNEHMPEDMFCHEFHLADLRVMDNCLKVTNGVDHVFNLAADMGGMGFIQSNHSVIMYNNTMISFNVLEASRINGVKRFFYASSACIYPEFKQLETNVSLKESDAWPAEPQDAYGLEKLATEELCKHYTKDFGIECRIGRFHNIYGPFGTWKGGREKAPAAFCRKALTSTDRFEMWGDGLQTRSFTFIDECVEGVLRLTKSDFREPVNIGSDEMVSMNEMAEIVLSFEDKKLPIHHIPGPEGVRGRNSDNTLIKEKLGWAPTMKLKDGLRITYFWIKDQIEKEKTQGVDLSIYGSSKVVGTQAPVQLGSLRAADGNEGL
