# Supplementary material for: Effect of light on ascorbic acid biosynthesis and bioinformatics analysis of related genes in Chinese chives
Source: PLoS One. 2024 Aug 22;19(8):e0307527. doi: 10.1371/journal.pone.0307527 (PMC11340962; doi:10.1371/journal.pone.0307527)
Supplement: S1 Fig — (PDF) [file pone.0307527.s004.pdf]

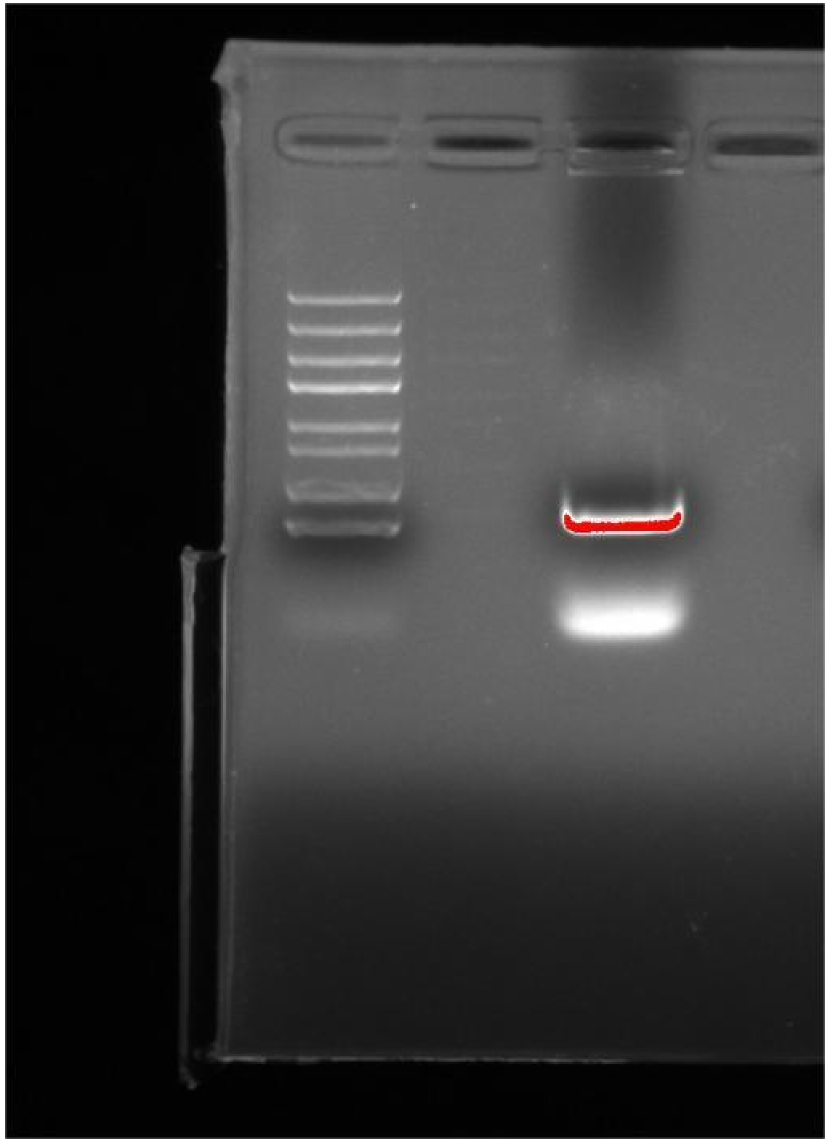

Fig 1. PCR electrophoretic of *AtuGGP1*

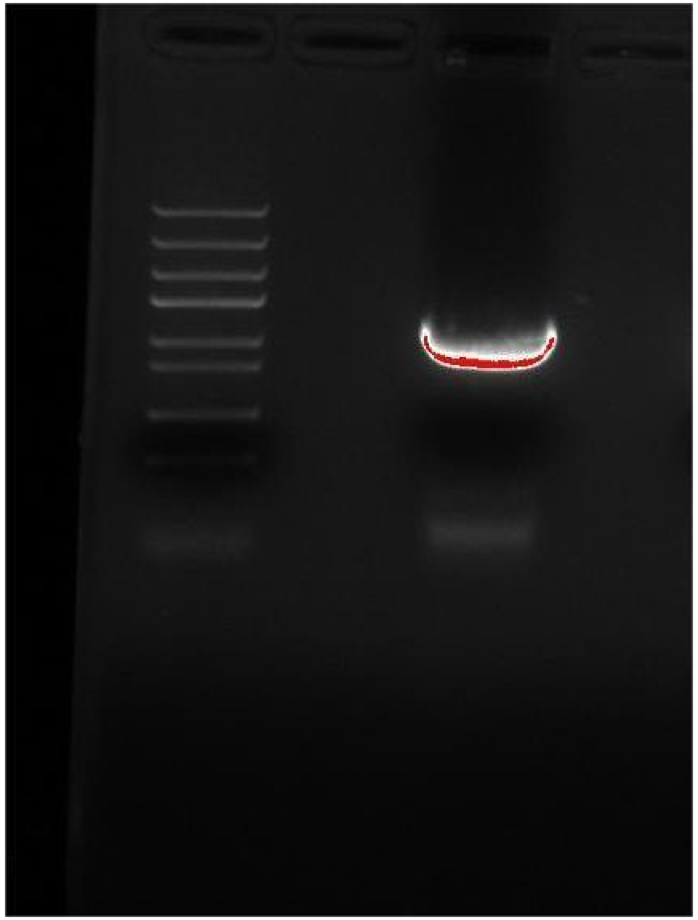

Fig 1. PCR electrophoretic of *AtuGME1*

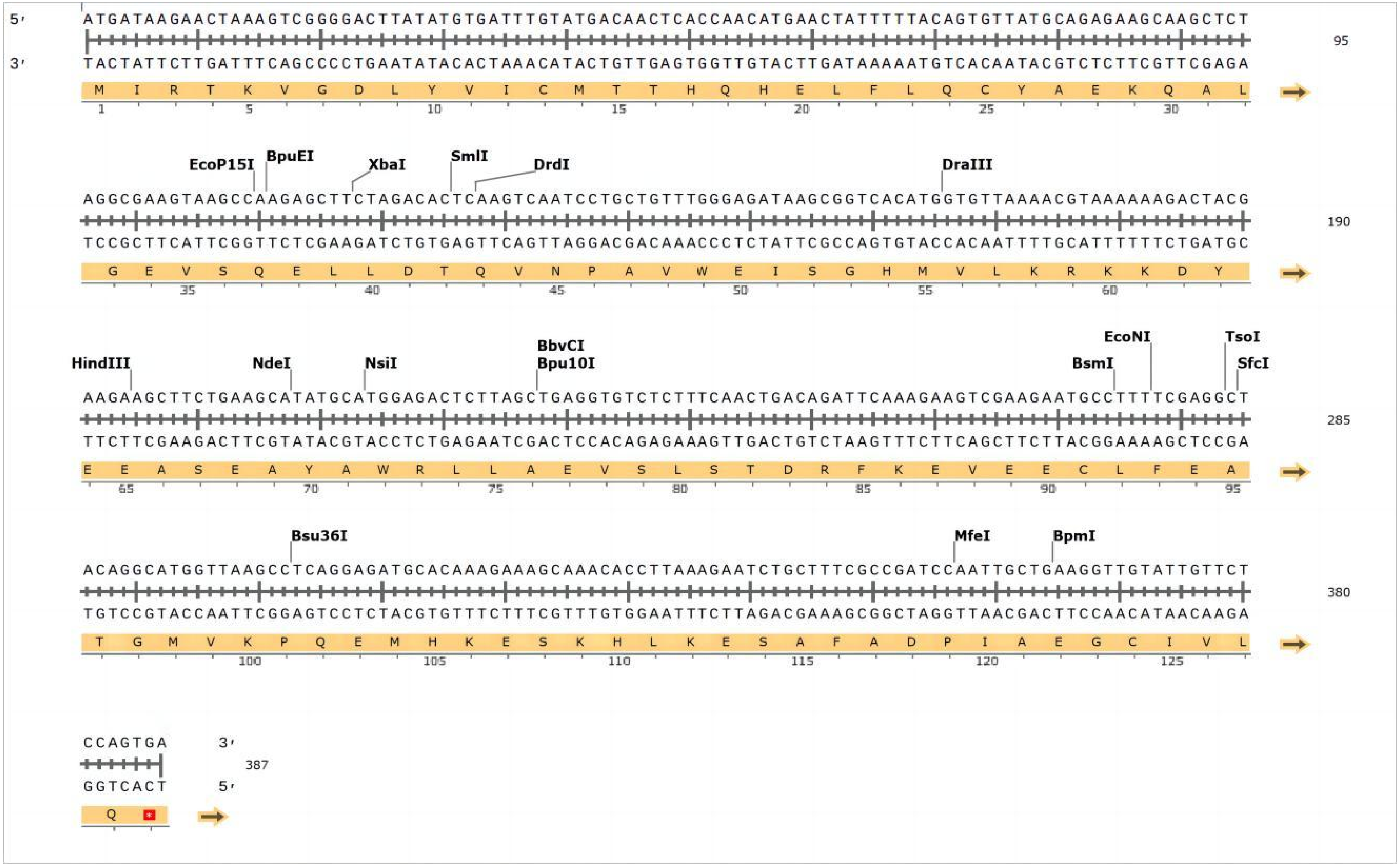

Fig 2. The deduced amino acid sequences of *AtuGGP1*

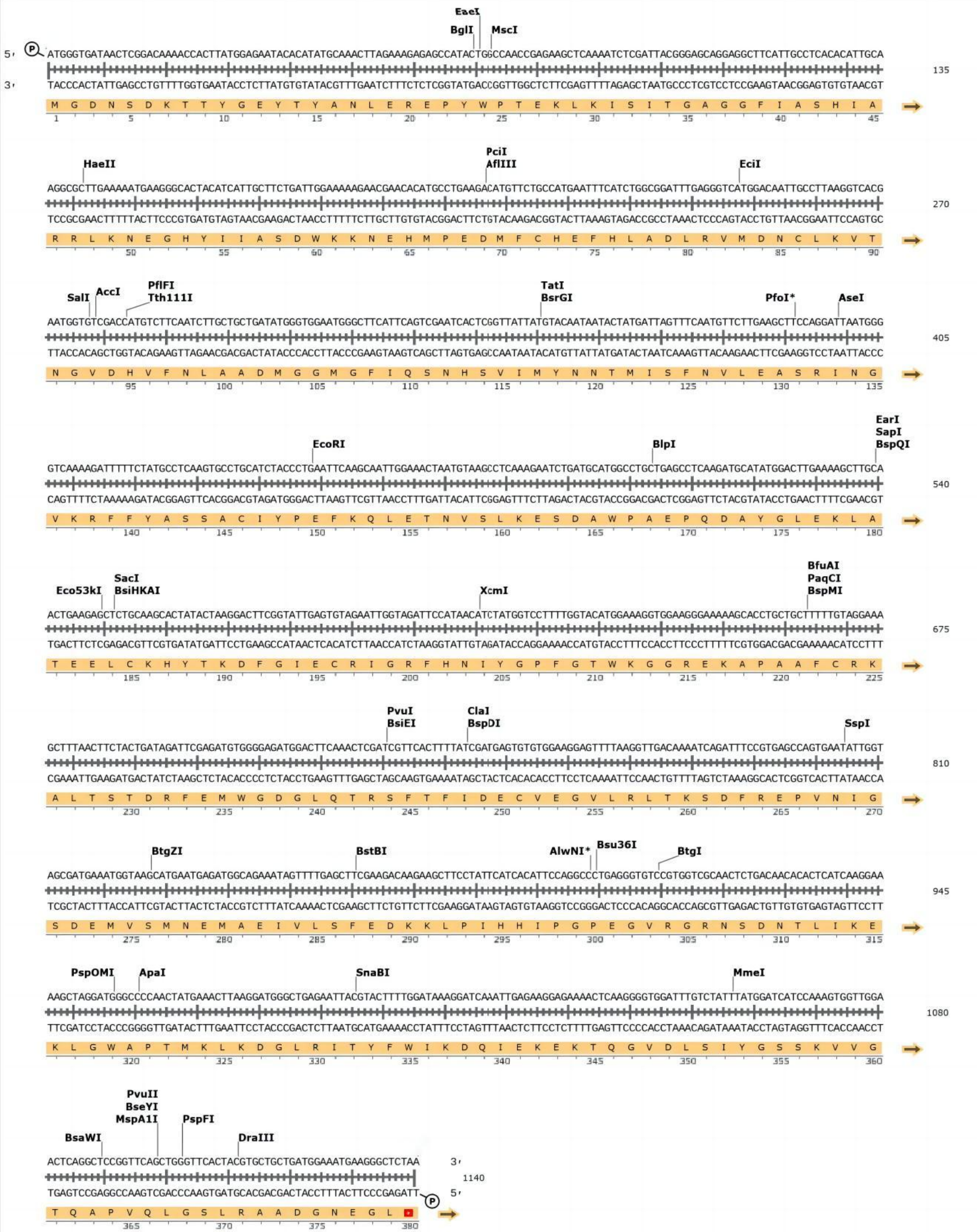

Fig 2. The deduced amino acid sequences of *AtuGME1*

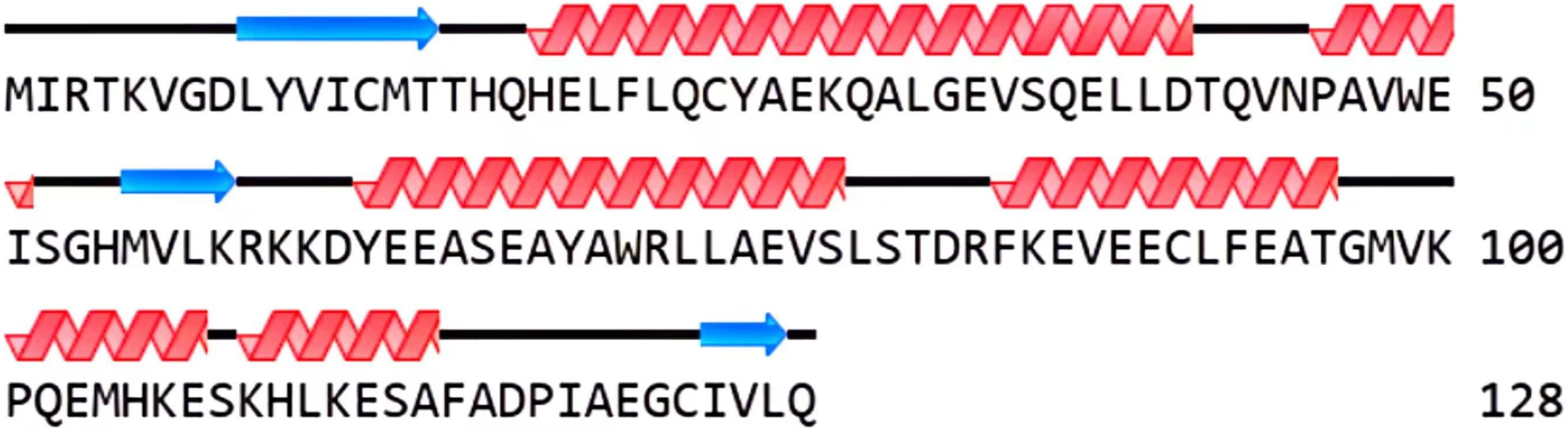

Fig 3. *AtuGGP1* secondary structure predictions

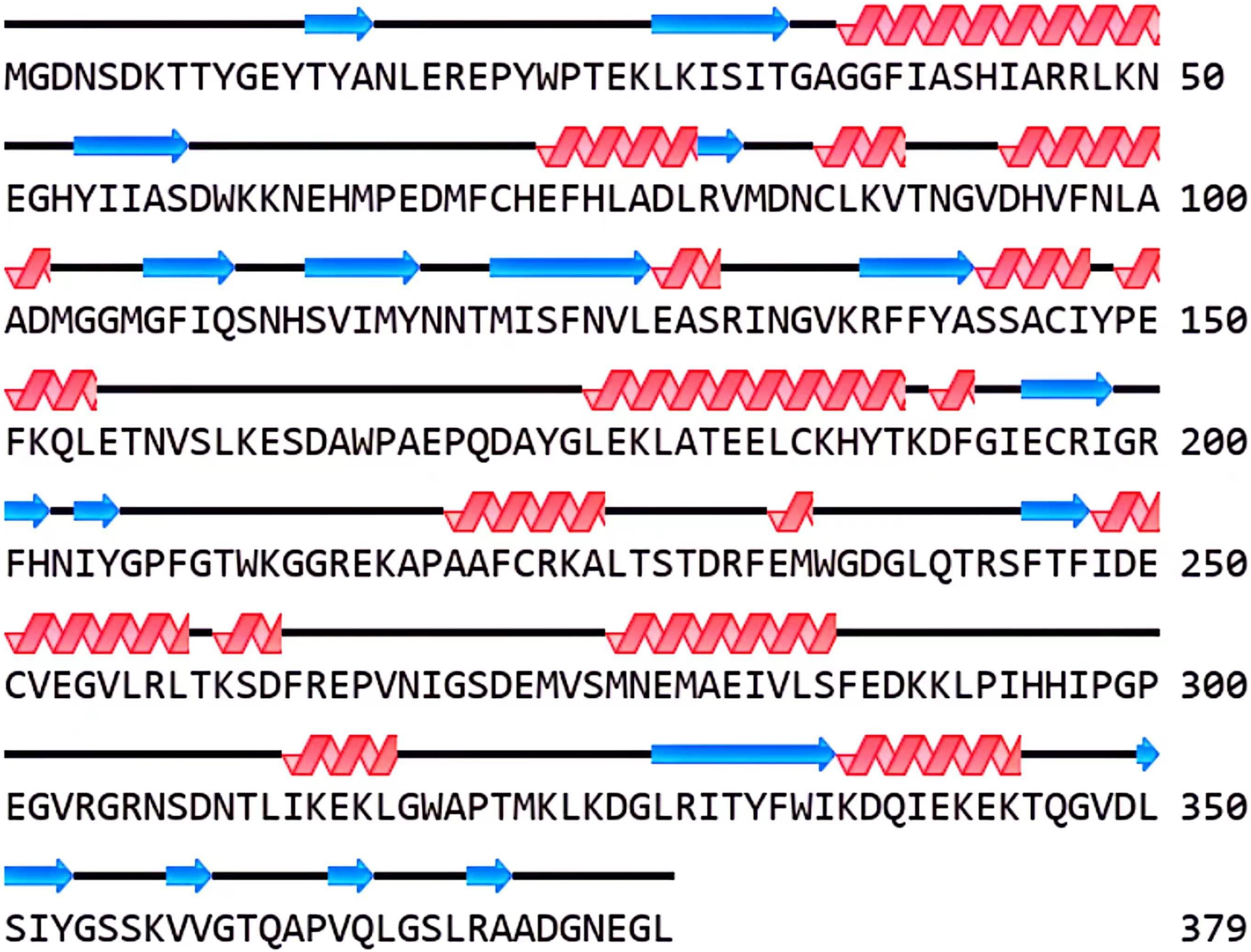

Fig 3. ***AtuGME1*** **secondary structure predictions**

Hydrophilicity Plot for user_sequence

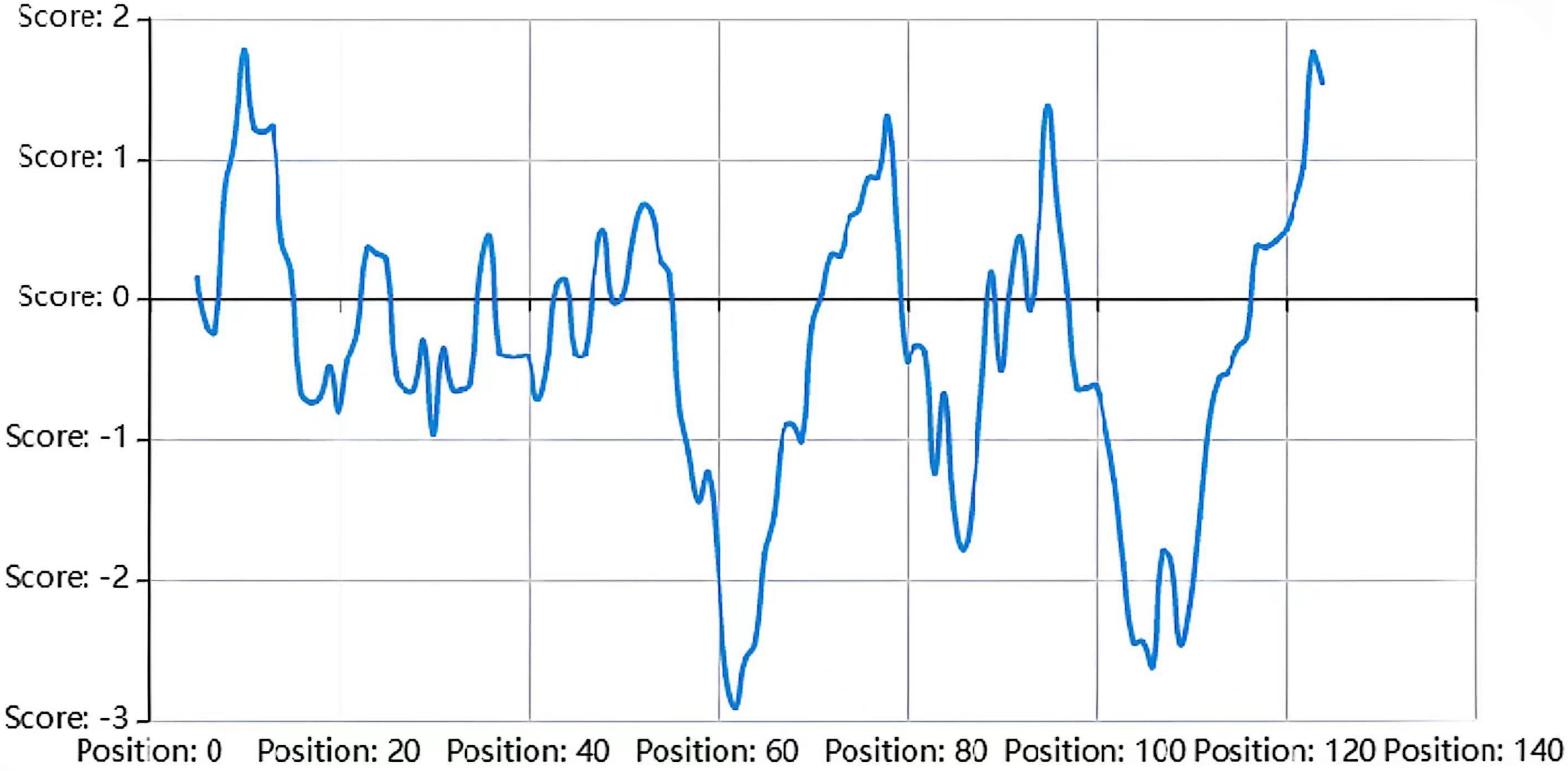

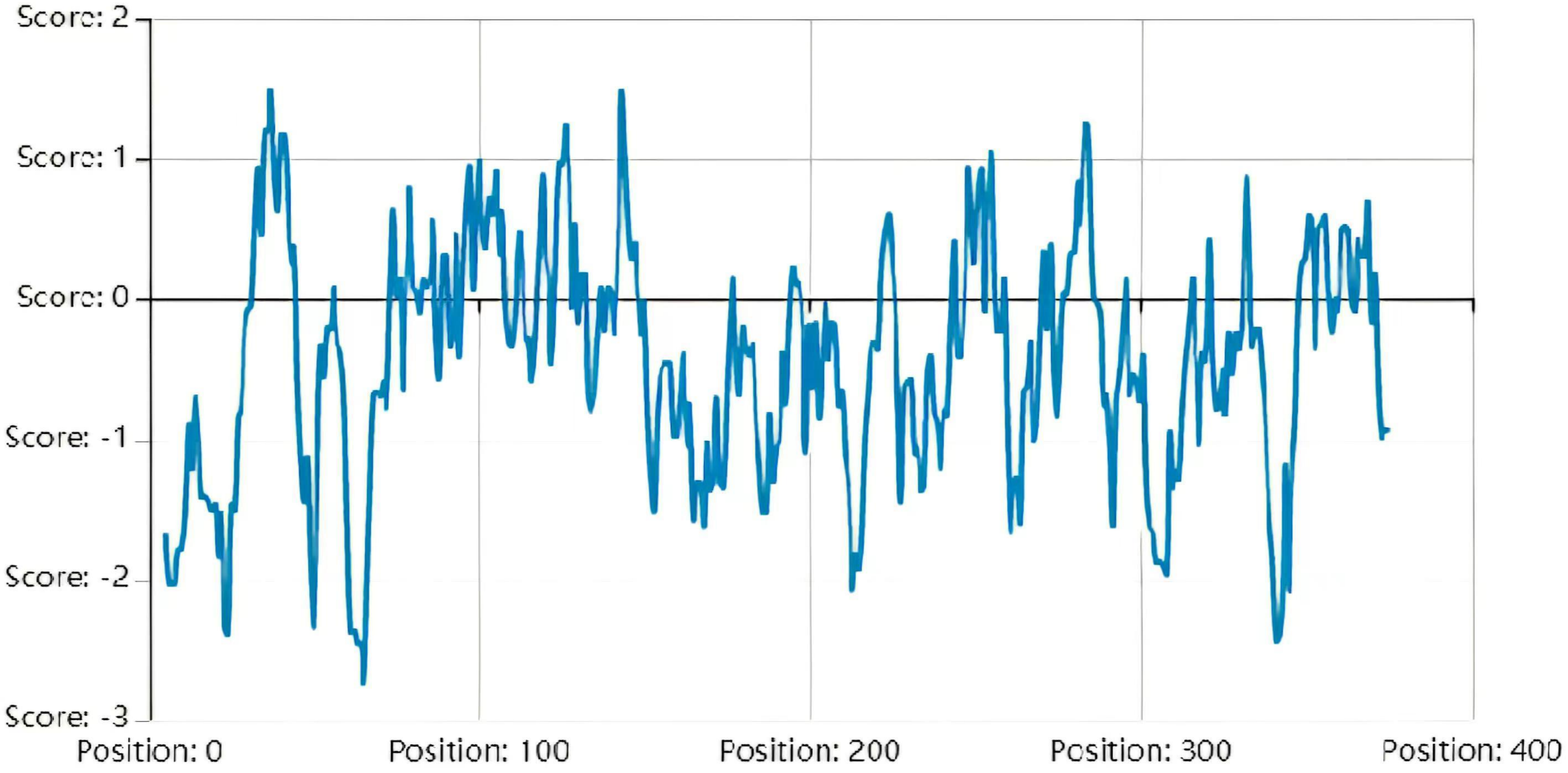
Score: 2
Score: 1
Score: 0
Score: -1
Score: -2
Score: -3
Position: 0
Position: 100
Position: 200
Position: 300
Position: 400

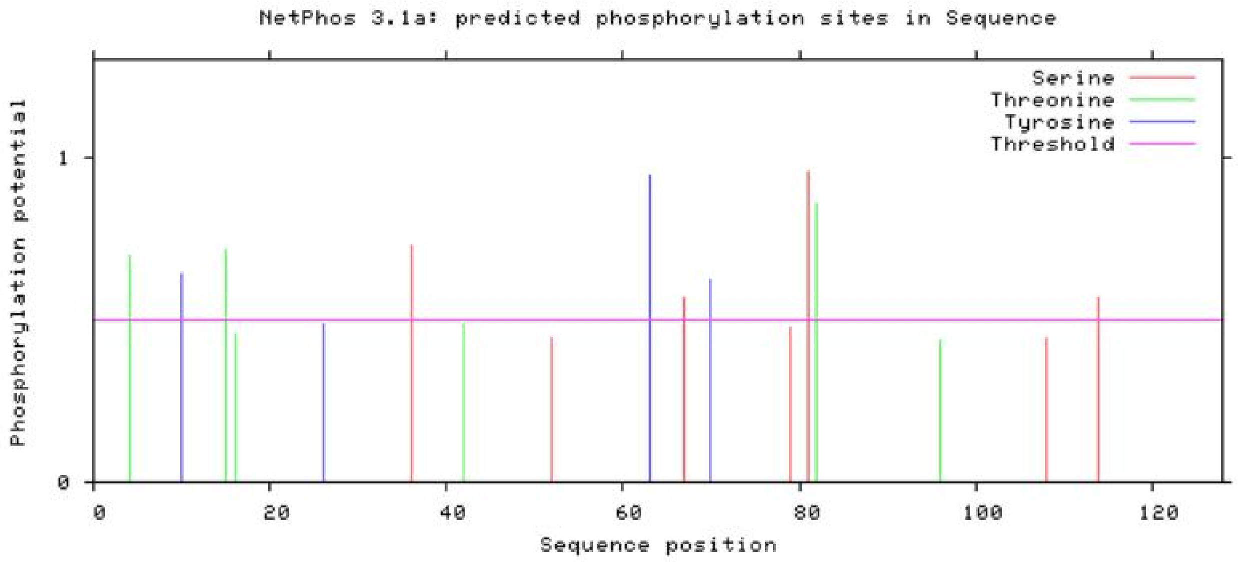

Fig 5. Protein phosphorylation site predictions in *AtuGGP1*

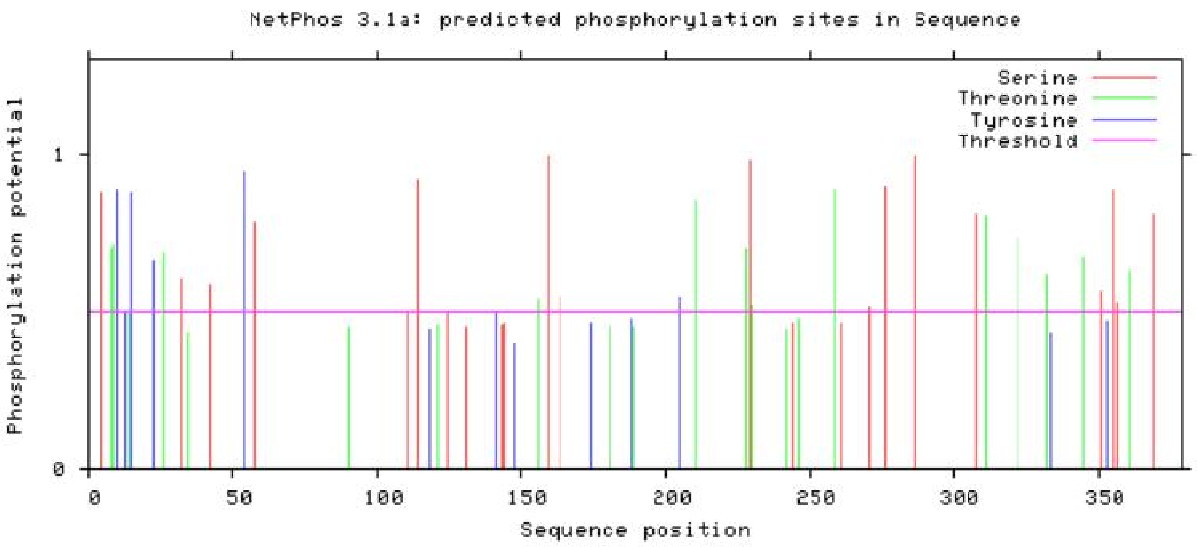

Fig 5. Protein phosphorylation site predictions in *AtuGME1*

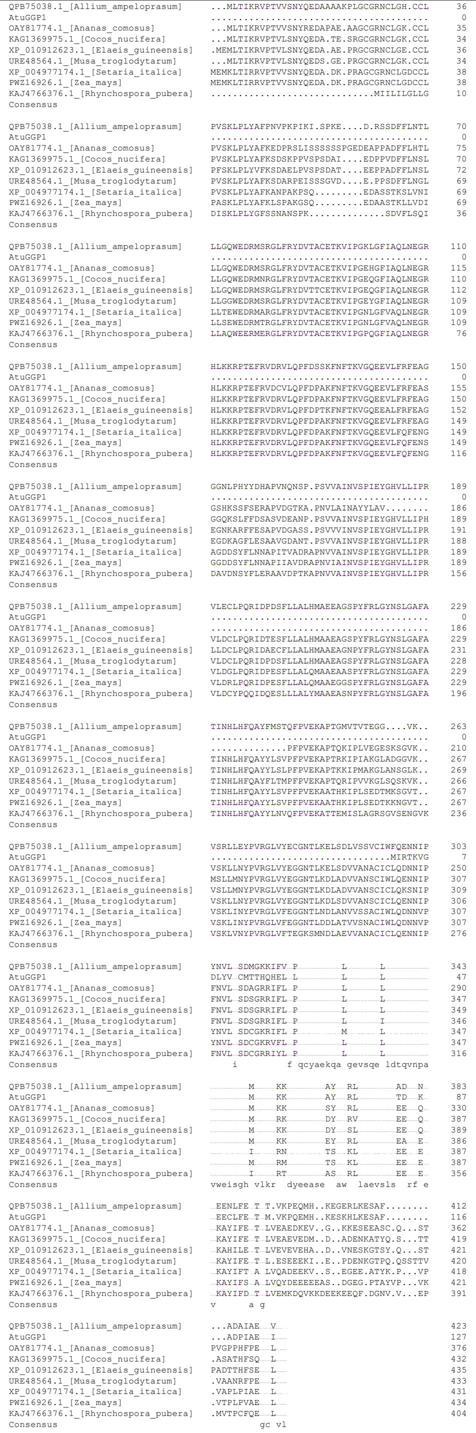

Fig 6. GGP amino acid sequence alignment of AtuGGP1 and other species

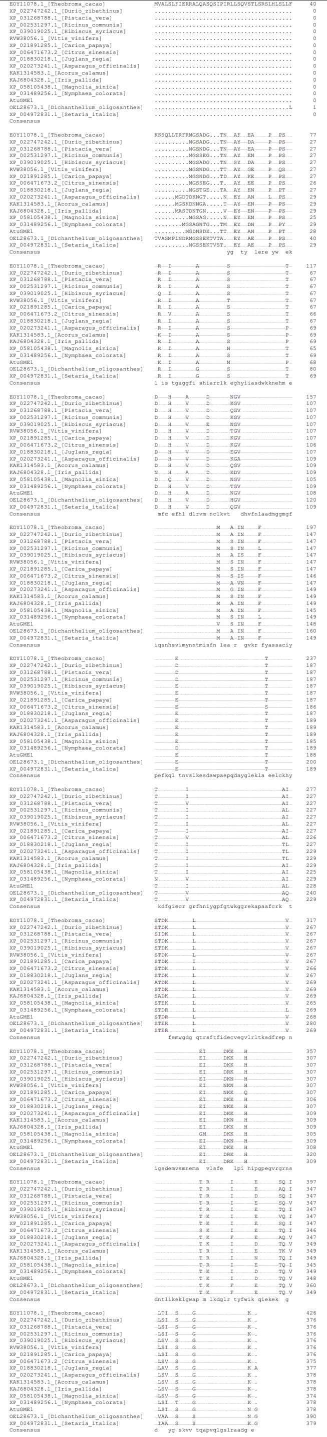

Fig 7. GME amino acid sequence alignment of AtuGME1 and other species

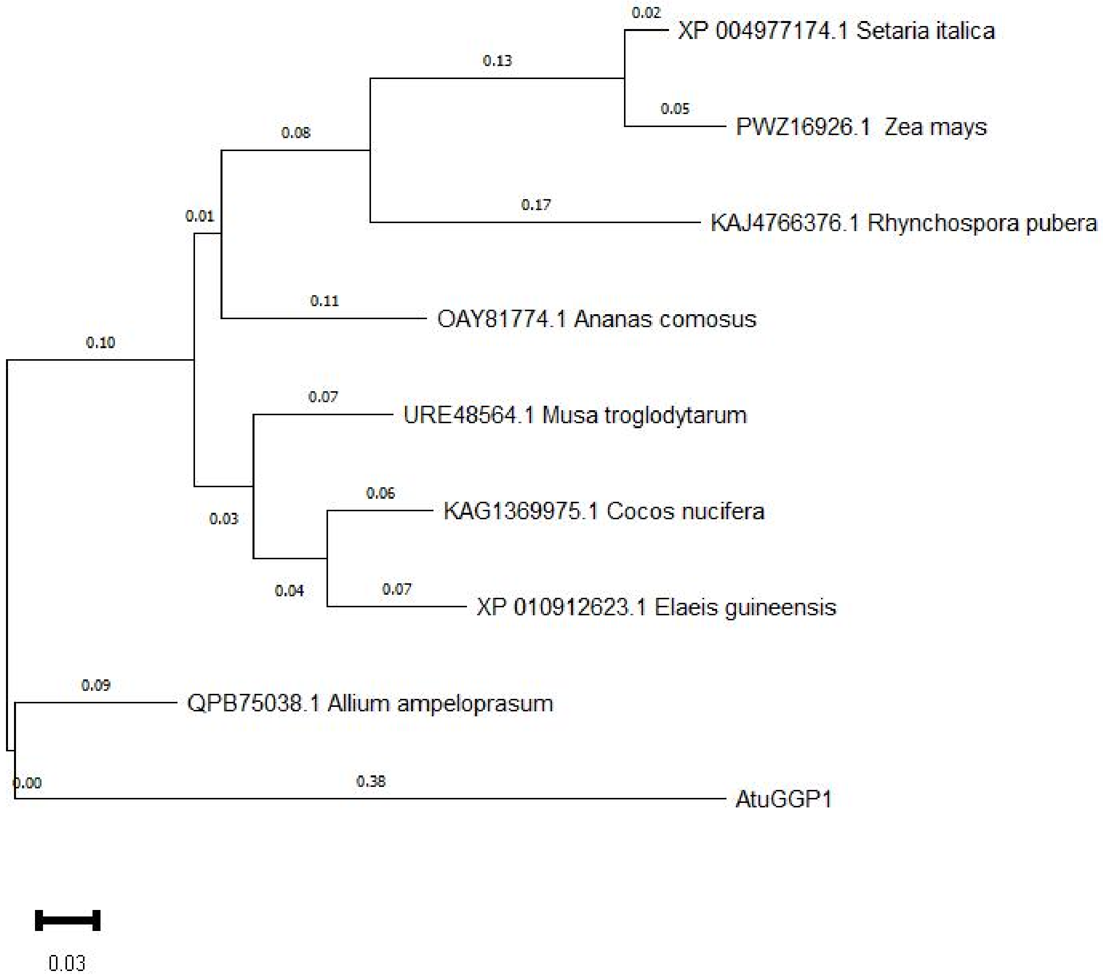

Fig 8. Phylogenetic trees of AtuGGP1

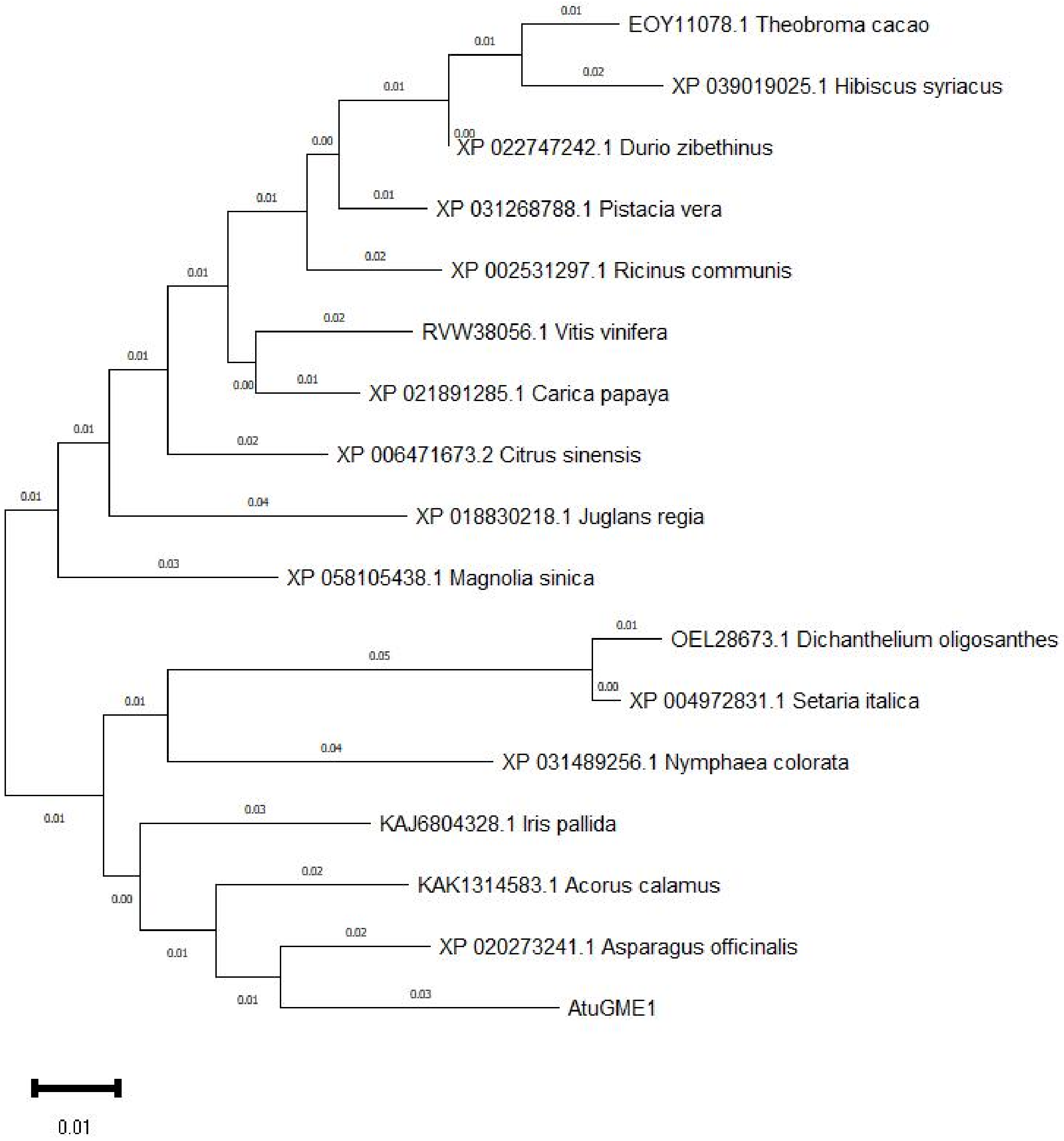

Fig 8. Phylogenetic trees of AtuGME1

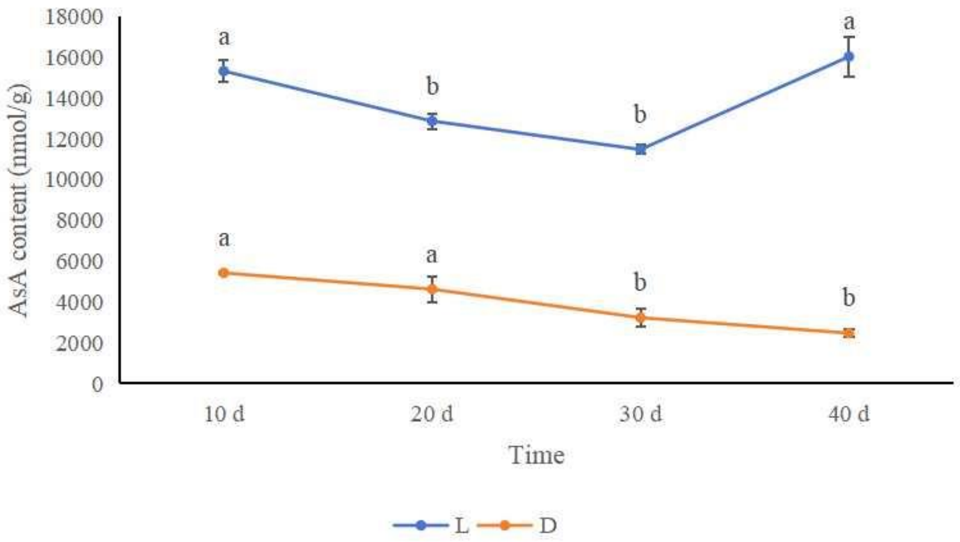

Fig 9. AsA content of Chinese chive. AsA content of chive grew in natural light (L) and continuous dark (D) during different growth periods.

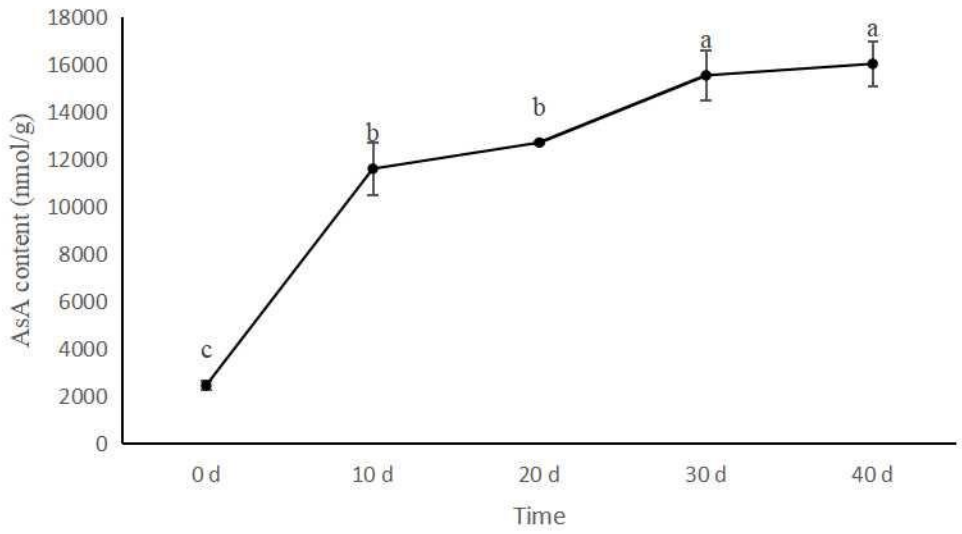

Fig 9. AsA content of Chinese chive. AsA content of chive grew under different proportions of lighting time.

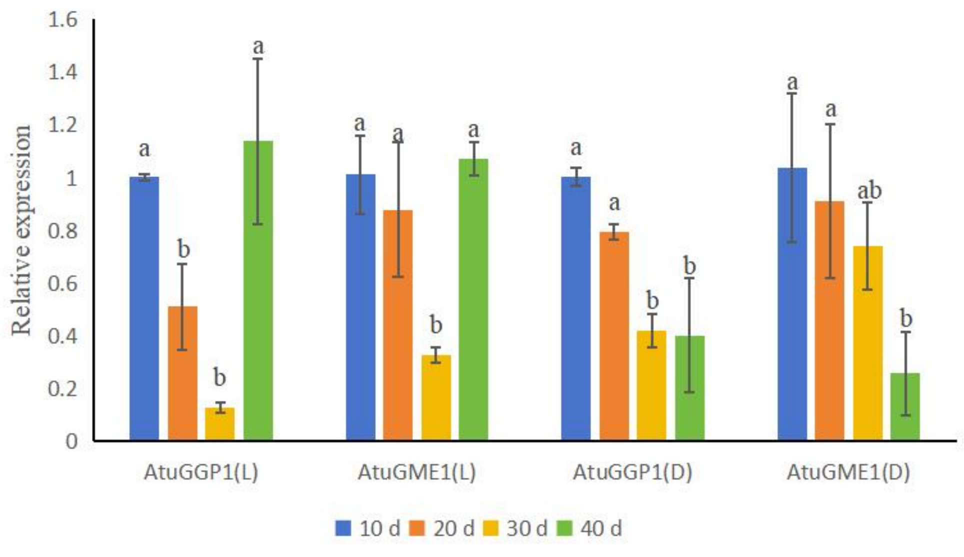

Fig 10. *AtuGGP1* and *AtuGME1* expression levels. *AtuGGP1* and *AtuGME1* expression levels of chive grew in natural light (L) and continuous dark (D) during different growth periods.

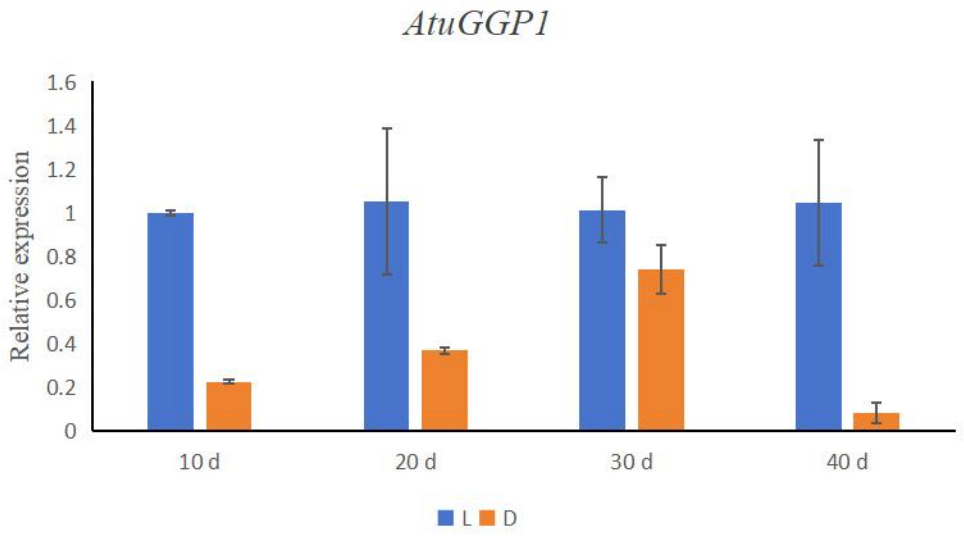

Fig 10. *AtuGGP1* and *AtuGME1* expression levels. L and D *AtuGGP1* relative expression levels during different growth periods.

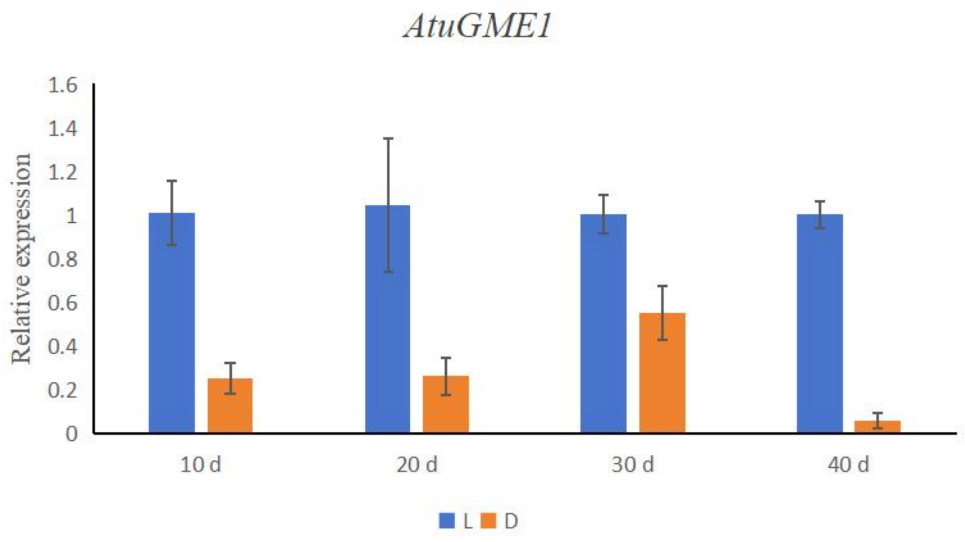

Fig 10. ***AtuGGP1*** **and** ***AtuGME1*** **expression levels.** L and D *AtuGME1* relative expression levels during different growth periods.

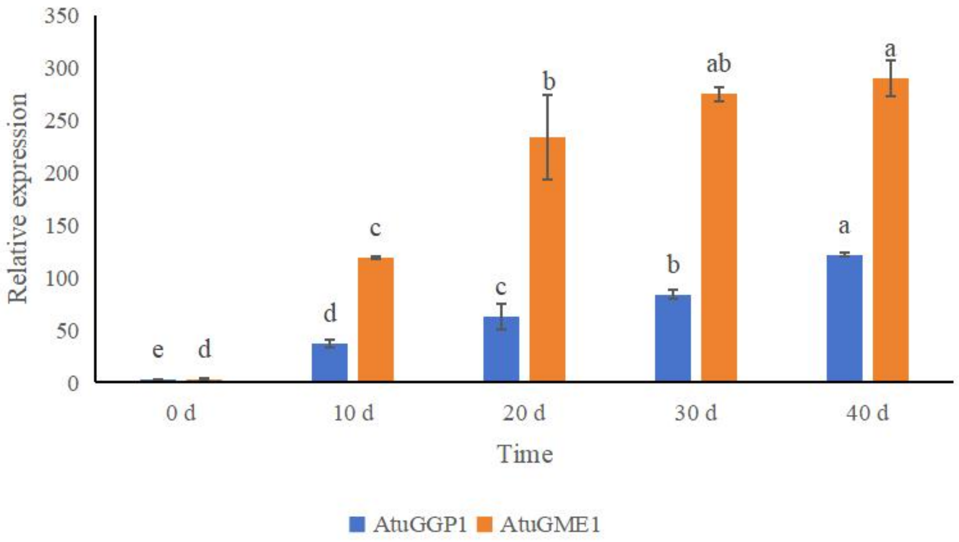

Fig 10. *AtuGGP1* and *AtuGME1* expression levels. *AtuGGP1* and *AtuGME1* expression levels of chive grew under different proportions of lighting time.
